# Supplementary figures and images for: Actin-related protein 5 functions as a novel modulator of MyoD and MyoG in skeletal muscle and in rhabdomyosarcoma (part 1 of 2)
Source: eLife. 2022 Mar 29;11:e77746. doi: 10.7554/eLife.77746 (PMC8983046; doi:10.7554/eLife.77746)

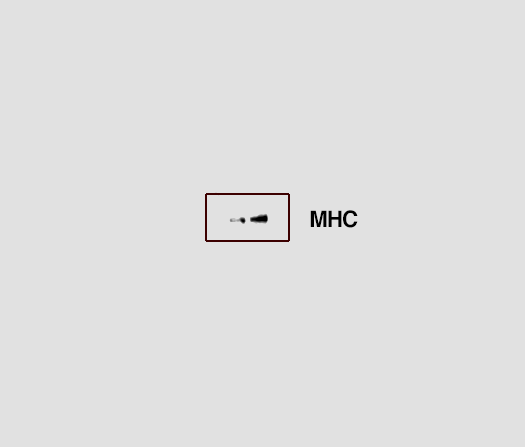

Supplement: Figure 1—source data 2. [file elife-77746-fig1-data2.zip › Fig. 1/+label/Fig.1B-MHC(+label).tif]

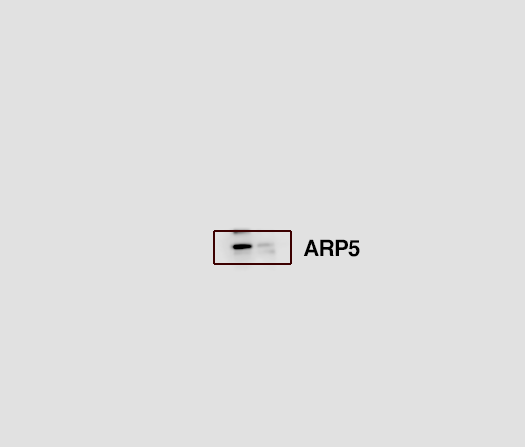

Supplement: Figure 1—source data 2. [file elife-77746-fig1-data2.zip › Fig. 1/+label/Fig.1B-Arp5(+label).tif]

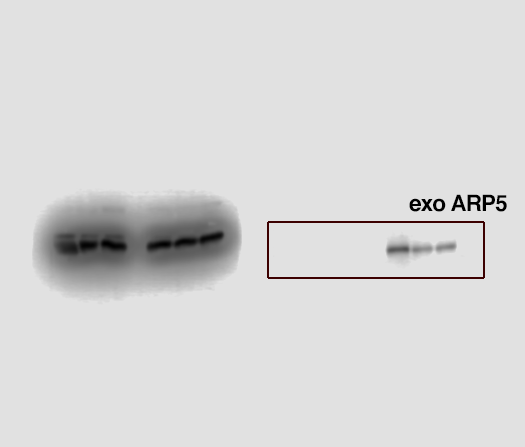

Supplement: Figure 1—source data 2. [file elife-77746-fig1-data2.zip › Fig. 1/+label/Fig.1F-exoArp5(+label).tif]

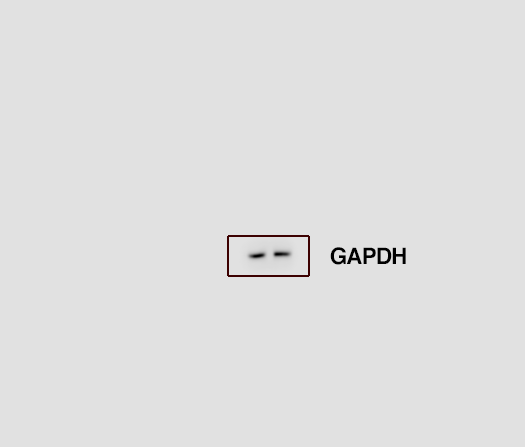

Supplement: Figure 1—source data 2. [file elife-77746-fig1-data2.zip › Fig. 1/+label/Fig.1B-GAPDH(+label).tif]

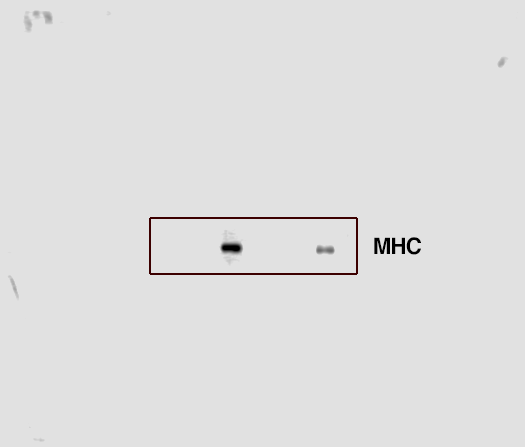

Supplement: Figure 1—source data 2. [file elife-77746-fig1-data2.zip › Fig. 1/+label/Fig.1F-MHC(+label).tif]

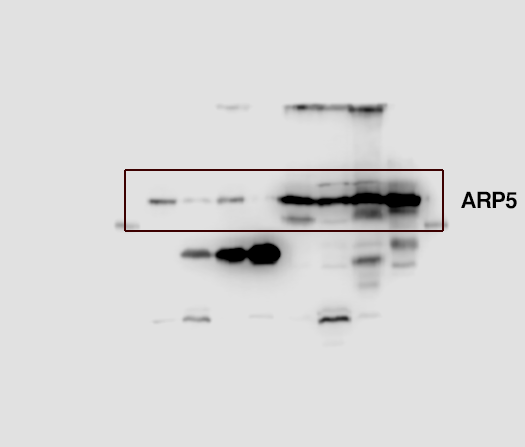

Supplement: Figure 1—source data 2. [file elife-77746-fig1-data2.zip › Fig. 1/+label/Fig.1A-Arp5(+label).tif]

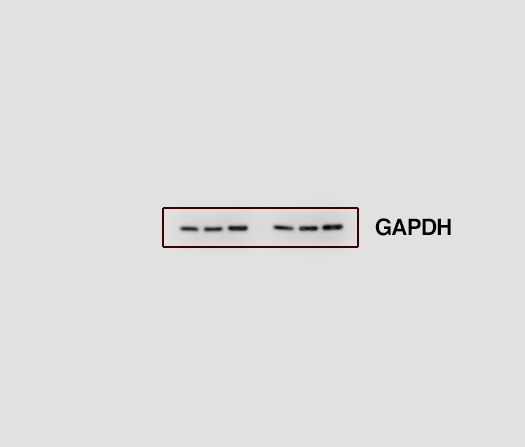

Supplement: Figure 1—source data 2. [file elife-77746-fig1-data2.zip › Fig. 1/+label/Fig.1F-GAPDH(+label).tif]

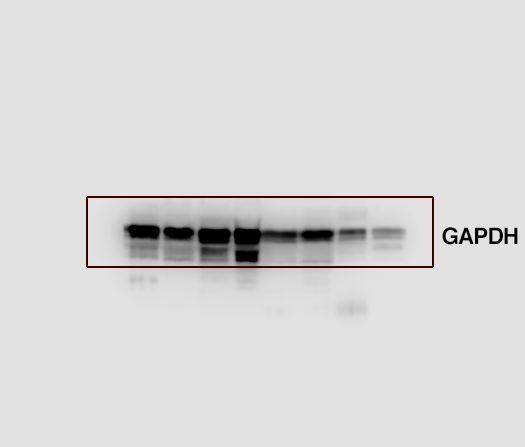

Supplement: Figure 1—source data 2. [file elife-77746-fig1-data2.zip › Fig. 1/+label/Fig.1A-GAPDH(+label).tif]

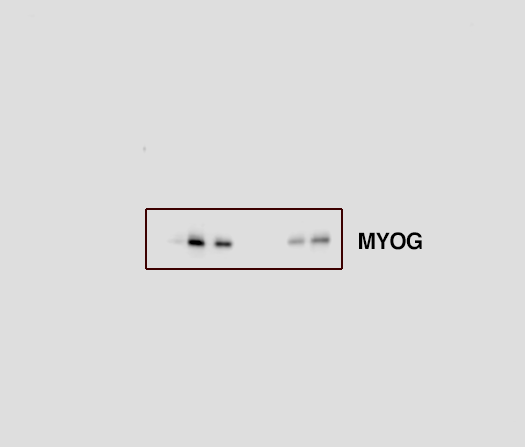

Supplement: Figure 1—source data 2. [file elife-77746-fig1-data2.zip › Fig. 1/+label/Fig.1F-MYOG(+label).tif]

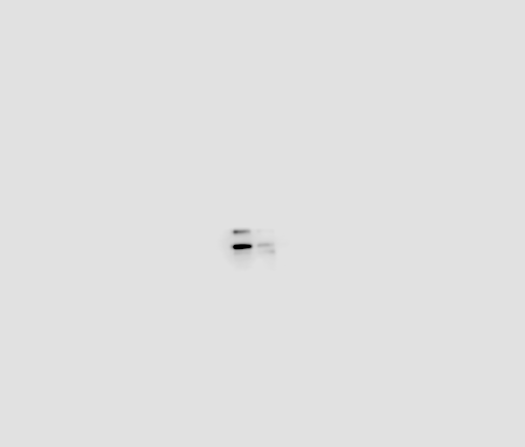

Supplement: Figure 1—source data 2. [file elife-77746-fig1-data2.zip › Fig. 1/unedited/Fig.1B-Arp5.tif]

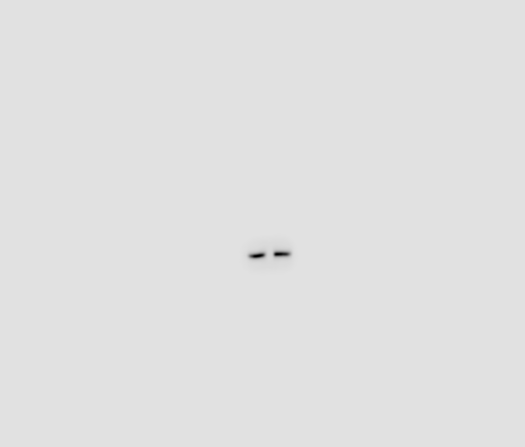

Supplement: Figure 1—source data 2. [file elife-77746-fig1-data2.zip › Fig. 1/unedited/Fig.1B-GAPDH.tif]

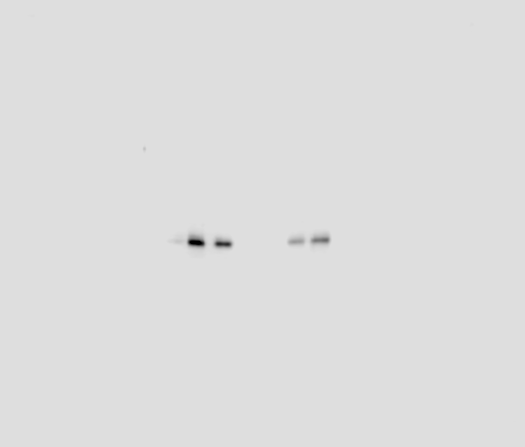

Supplement: Figure 1—source data 2. [file elife-77746-fig1-data2.zip › Fig. 1/unedited/Fig.1F-MYOG.tif]

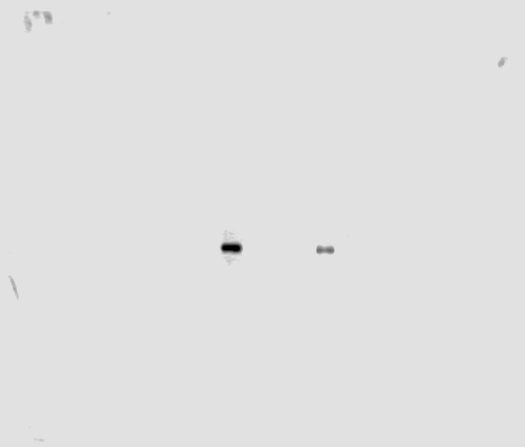

Supplement: Figure 1—source data 2. [file elife-77746-fig1-data2.zip › Fig. 1/unedited/Fig.1F-MHC.tif]

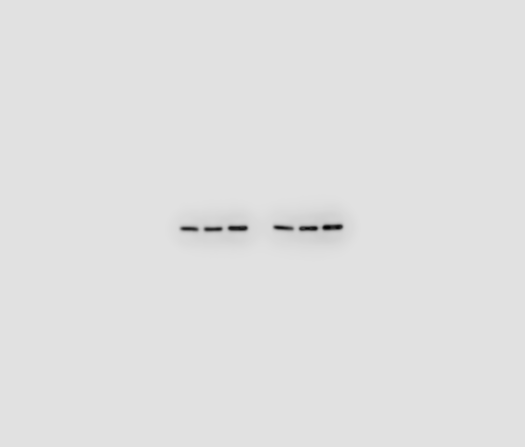

Supplement: Figure 1—source data 2. [file elife-77746-fig1-data2.zip › Fig. 1/unedited/Fig.1F-GAPDH.tif]

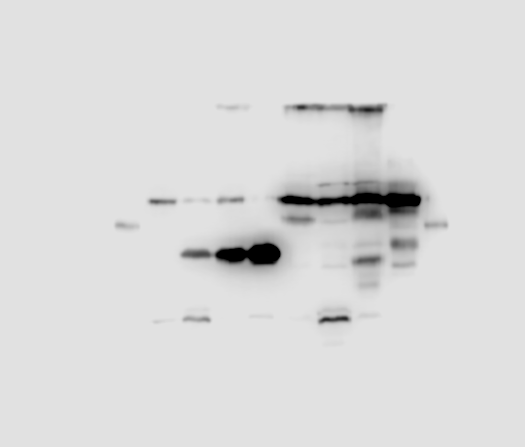

Supplement: Figure 1—source data 2. [file elife-77746-fig1-data2.zip › Fig. 1/unedited/Fig.1A-Arp5.tif]

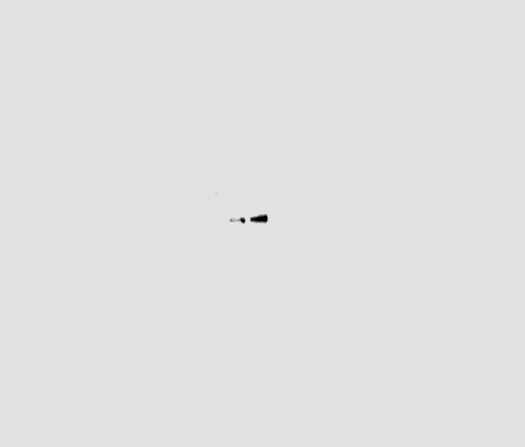

Supplement: Figure 1—source data 2. [file elife-77746-fig1-data2.zip › Fig. 1/unedited/Fig.1B-MHC.tif]

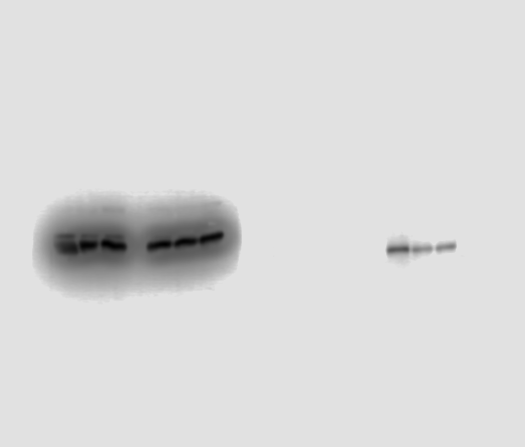

Supplement: Figure 1—source data 2. [file elife-77746-fig1-data2.zip › Fig. 1/unedited/Fig.1F-exoArp5.tif]

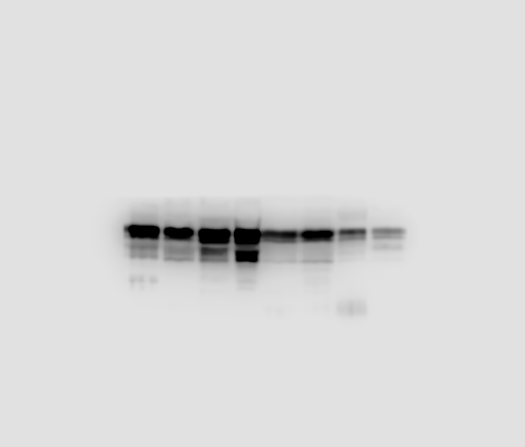

Supplement: Figure 1—source data 2. [file elife-77746-fig1-data2.zip › Fig. 1/unedited/Fig.1A-GAPDH.tif]

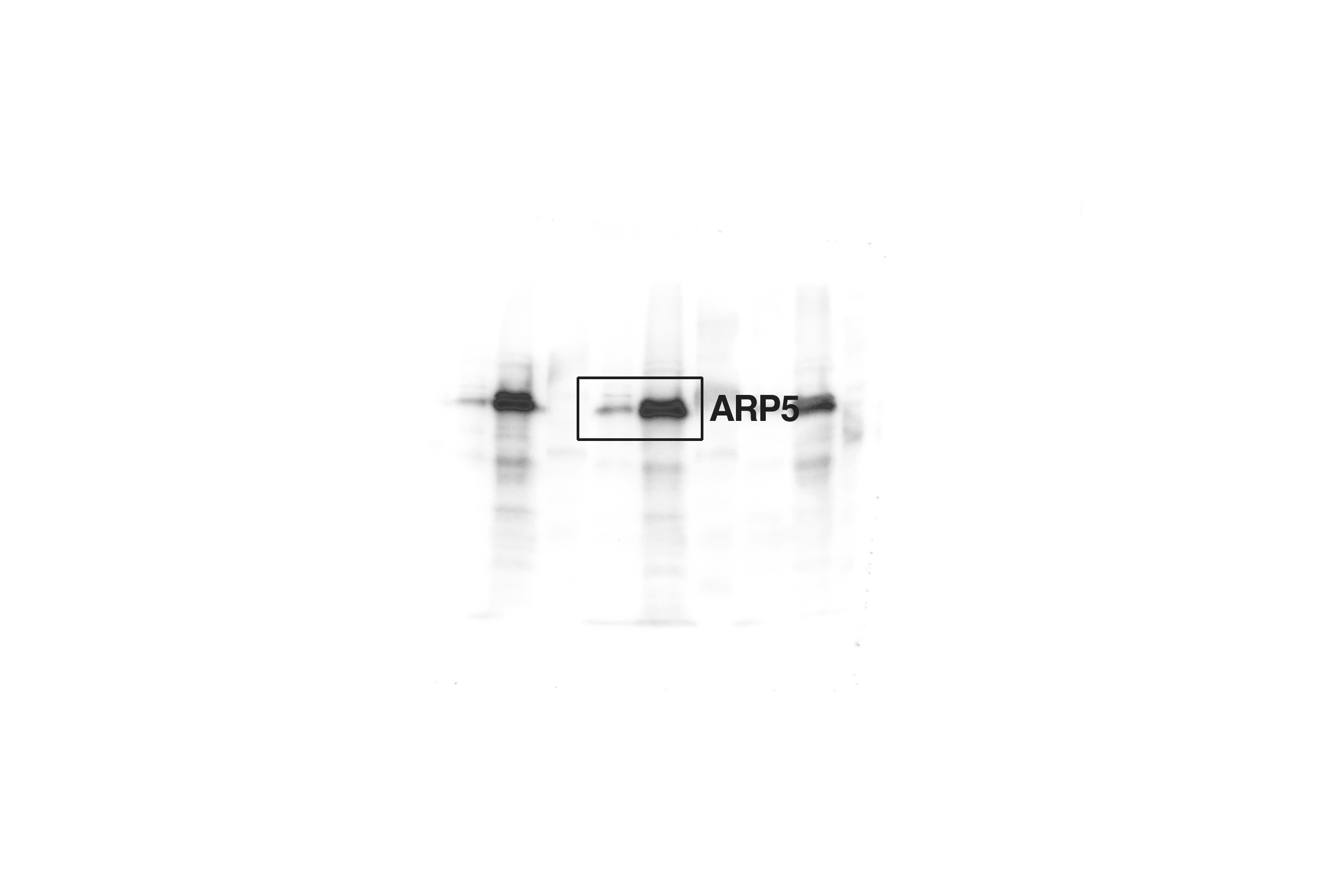

Supplement: Figure 2—source data 2. [file elife-77746-fig2-data2.zip › Fig.2/+label/Fig.2B-ARP5(+label).tif]

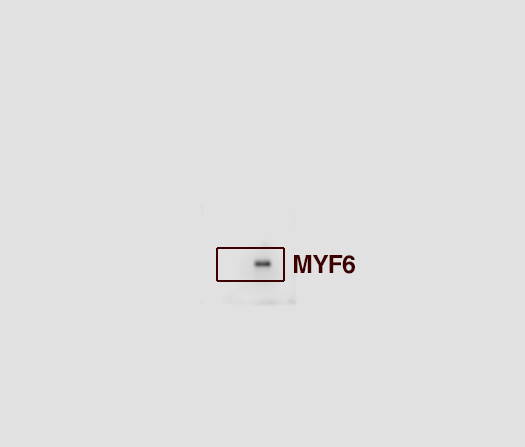

Supplement: Figure 2—source data 2. [file elife-77746-fig2-data2.zip › Fig.2/+label/Fig.2F-MYF6(+label).tif]

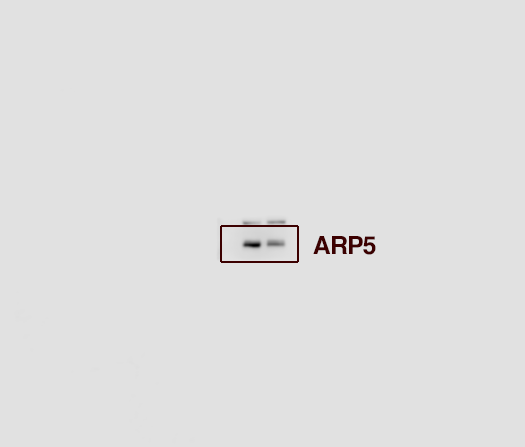

Supplement: Figure 2—source data 2. [file elife-77746-fig2-data2.zip › Fig.2/+label/Fig.2F-Arp5(+label).tif]

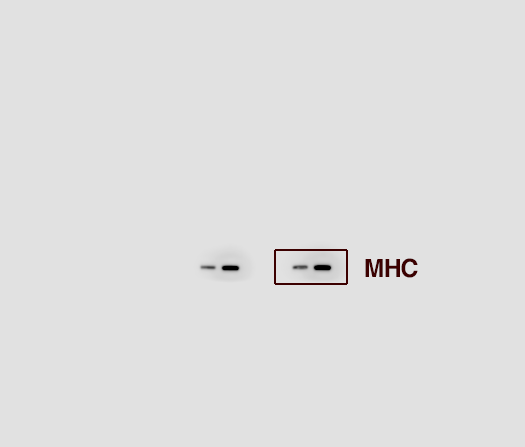

Supplement: Figure 2—source data 2. [file elife-77746-fig2-data2.zip › Fig.2/+label/Fig.2F-MHC(+label).tif]

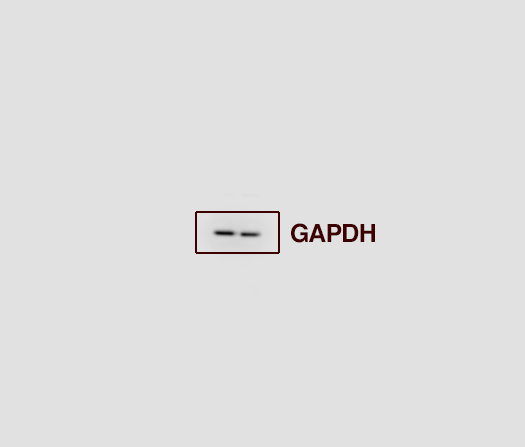

Supplement: Figure 2—source data 2. [file elife-77746-fig2-data2.zip › Fig.2/+label/Fig.2F-GAPDH(+label).tif]

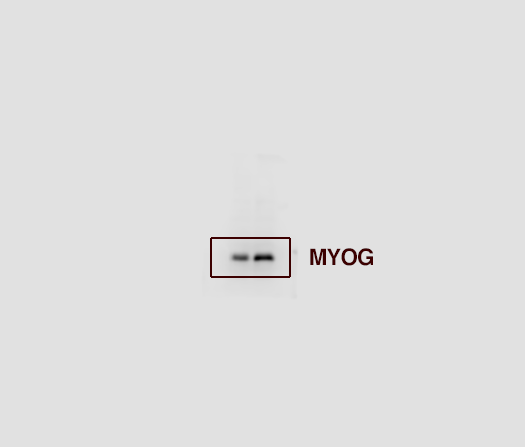

Supplement: Figure 2—source data 2. [file elife-77746-fig2-data2.zip › Fig.2/+label/Fig.2F-MyoG(+label).tif]

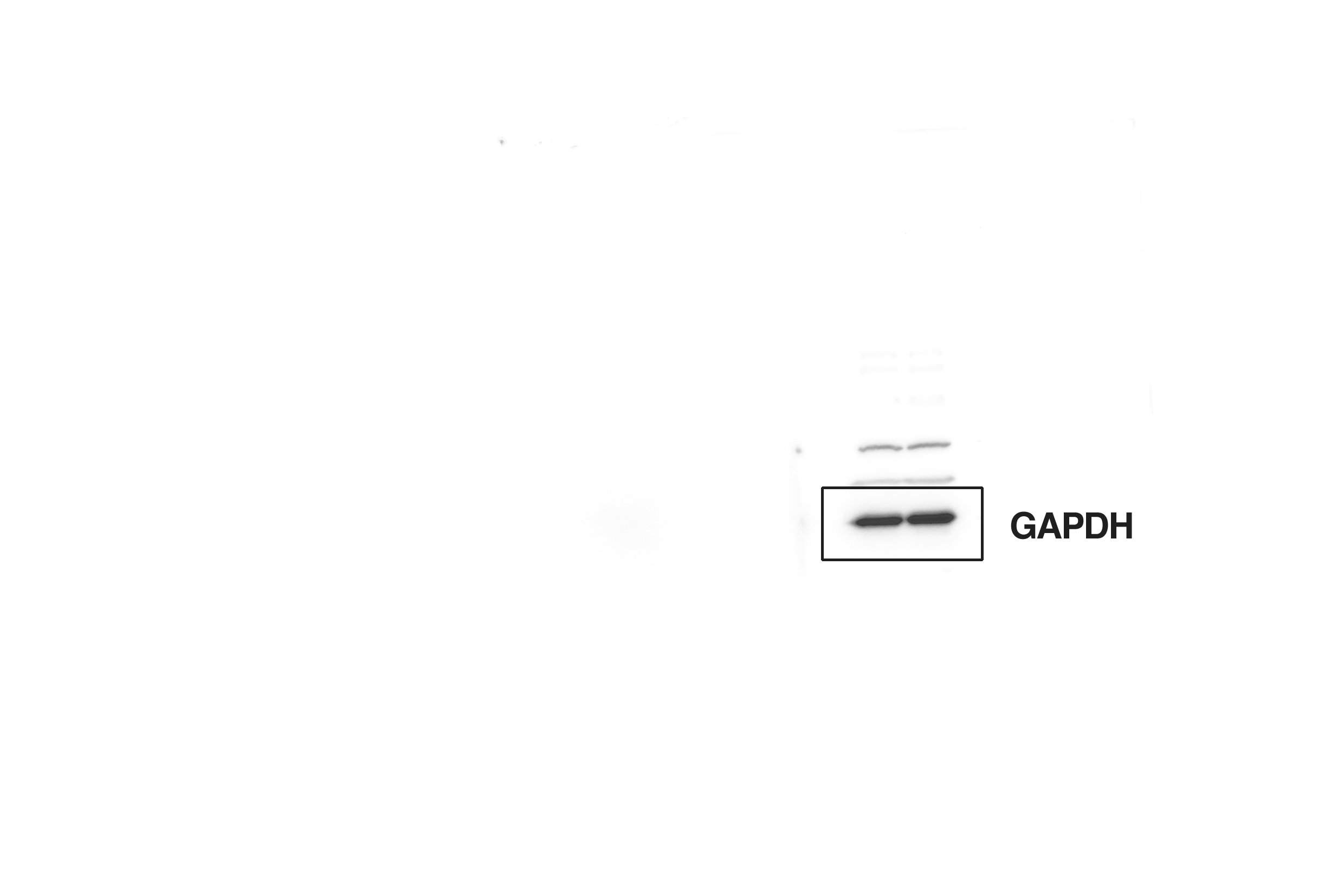

Supplement: Figure 2—source data 2. [file elife-77746-fig2-data2.zip › Fig.2/+label/Fig.2B-GAPDH(+label).tif]

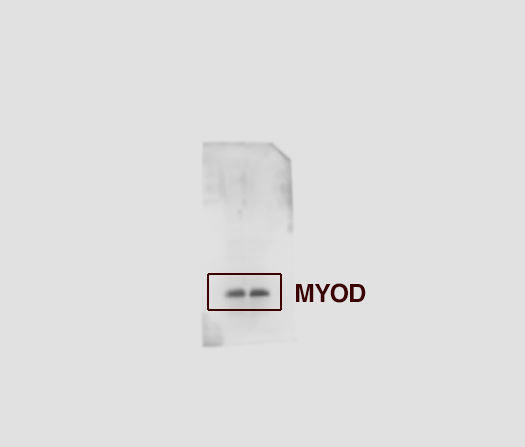

Supplement: Figure 2—source data 2. [file elife-77746-fig2-data2.zip › Fig.2/+label/Fig.2F-MyoD(+label).tif]

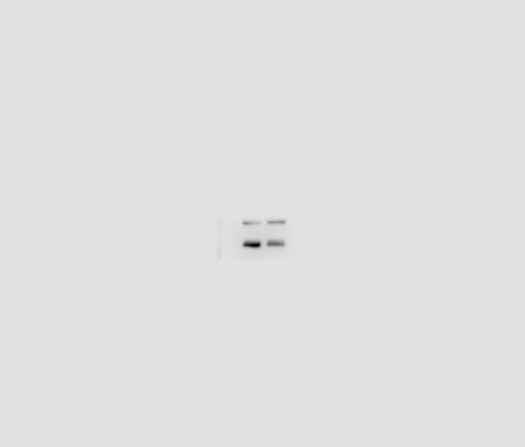

Supplement: Figure 2—source data 2. [file elife-77746-fig2-data2.zip › Fig.2/uneditied/Fig.2F-Arp5.tif]

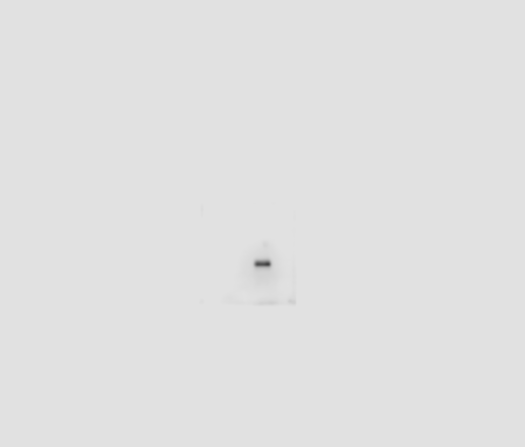

Supplement: Figure 2—source data 2. [file elife-77746-fig2-data2.zip › Fig.2/uneditied/Fig.2F-MYF6.tif]

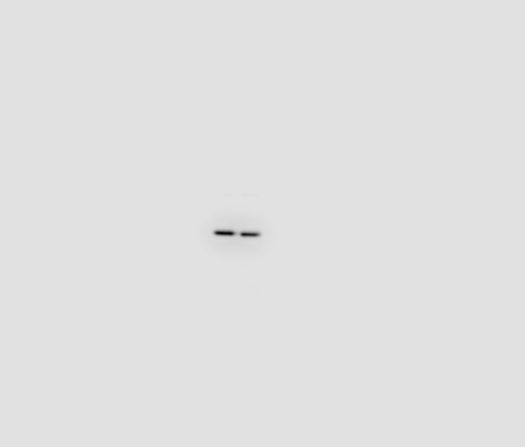

Supplement: Figure 2—source data 2. [file elife-77746-fig2-data2.zip › Fig.2/uneditied/Fig.2F-GAPDH.tif]

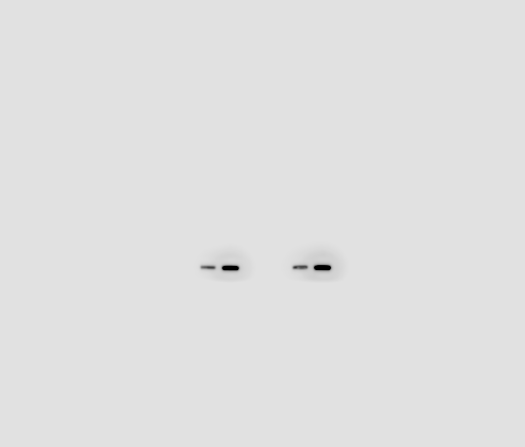

Supplement: Figure 2—source data 2. [file elife-77746-fig2-data2.zip › Fig.2/uneditied/Fig.2F-MHC.tif]

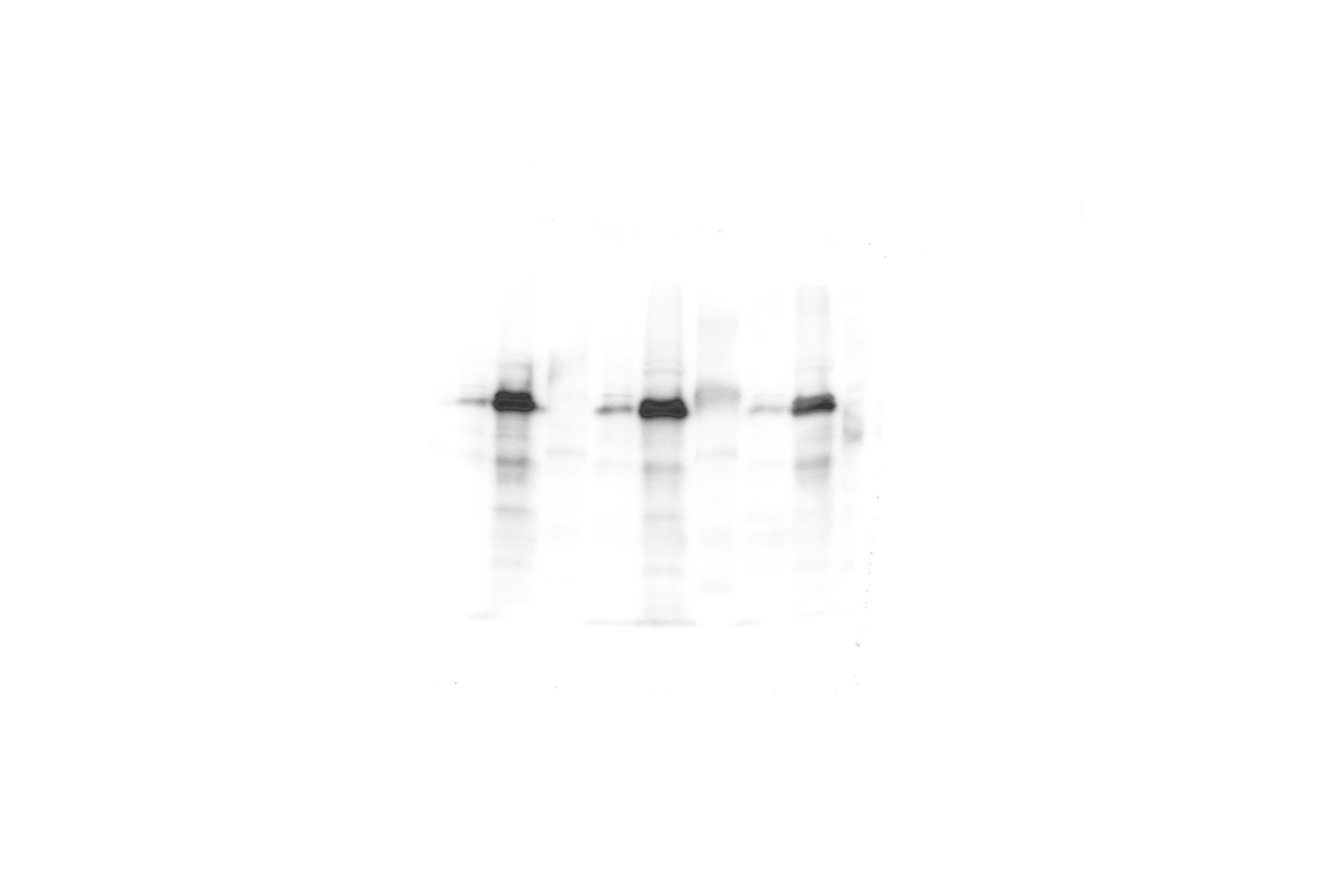

Supplement: Figure 2—source data 2. [file elife-77746-fig2-data2.zip › Fig.2/uneditied/Fig.2B-ARP5.tif]

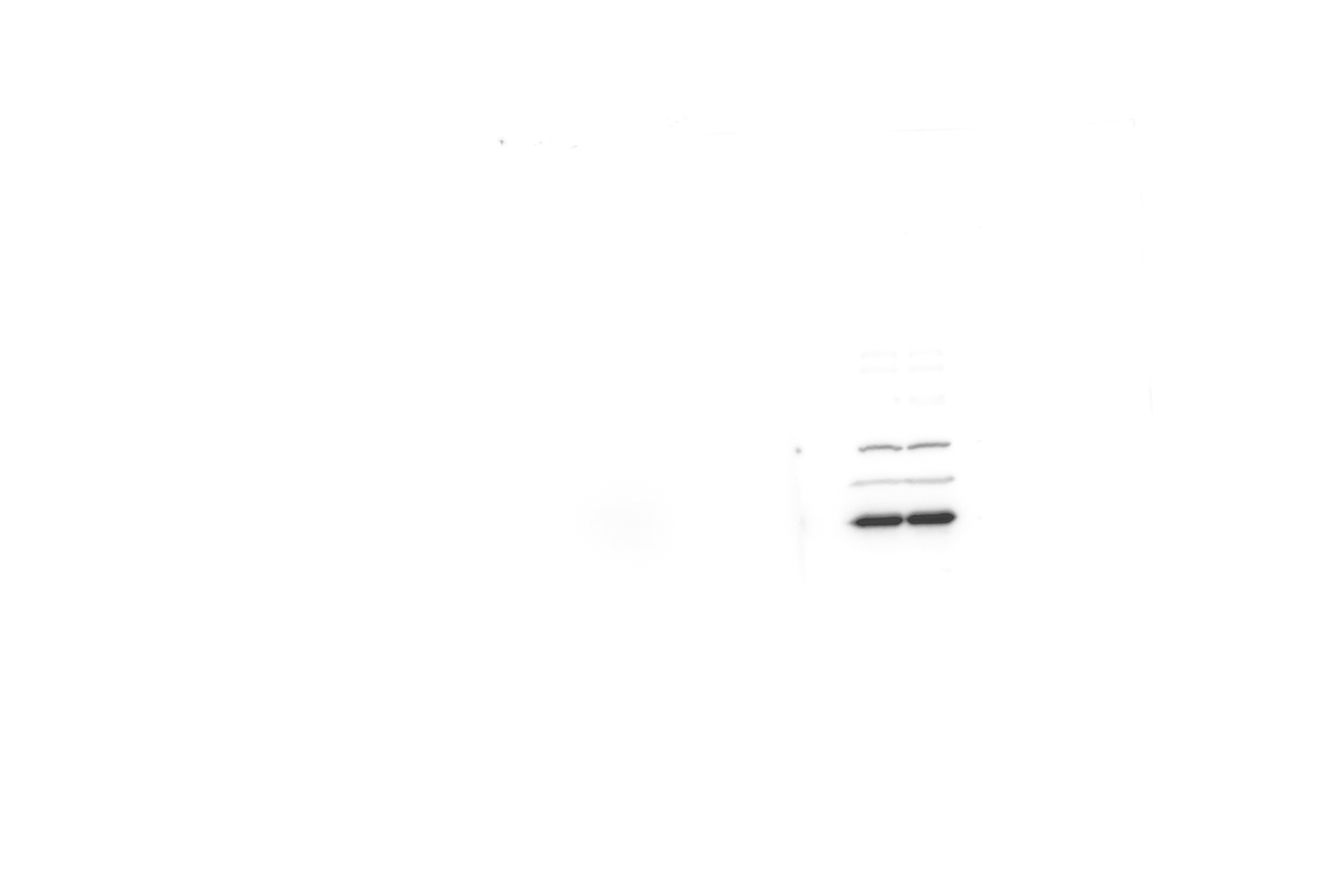

Supplement: Figure 2—source data 2. [file elife-77746-fig2-data2.zip › Fig.2/uneditied/Fig.2B-GAPDH.tif]

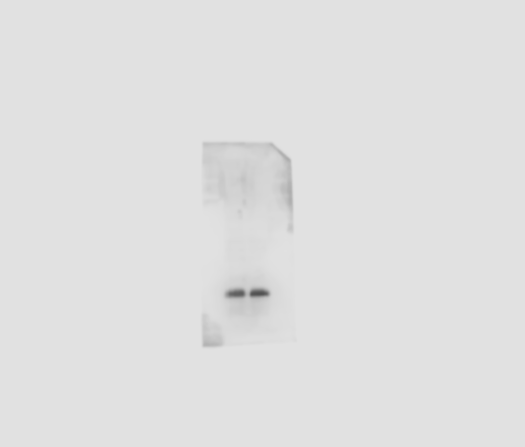

Supplement: Figure 2—source data 2. [file elife-77746-fig2-data2.zip › Fig.2/uneditied/Fig.2F-MyoD.tif]

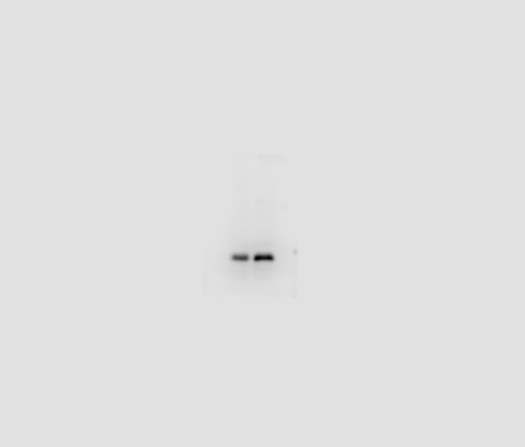

Supplement: Figure 2—source data 2. [file elife-77746-fig2-data2.zip › Fig.2/uneditied/Fig.2F-MyoG.tif]

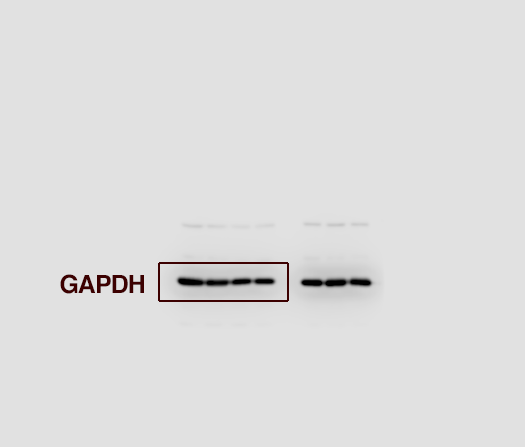

Supplement: Figure 3—source data 2. [file elife-77746-fig3-data2.zip › Fig.3/+label/Fig.3A-GAPDH(+label).tif]

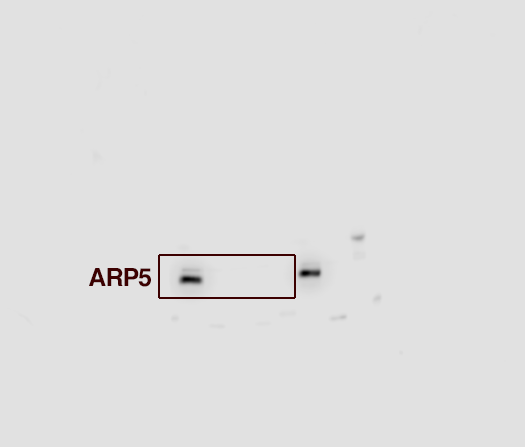

Supplement: Figure 3—source data 2. [file elife-77746-fig3-data2.zip › Fig.3/+label/Fig.3A-ARP5(+label).tif]

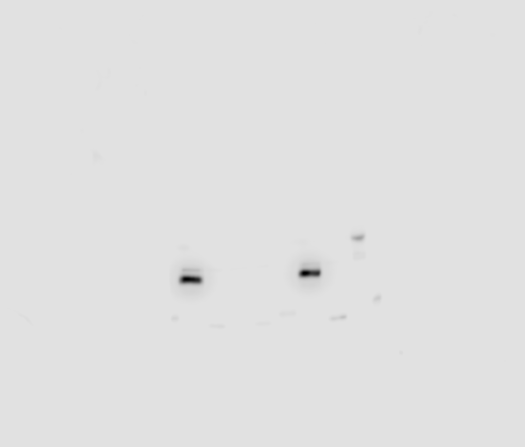

Supplement: Figure 3—source data 2. [file elife-77746-fig3-data2.zip › Fig.3/unedited/Fig.3A-ARP5.tif]

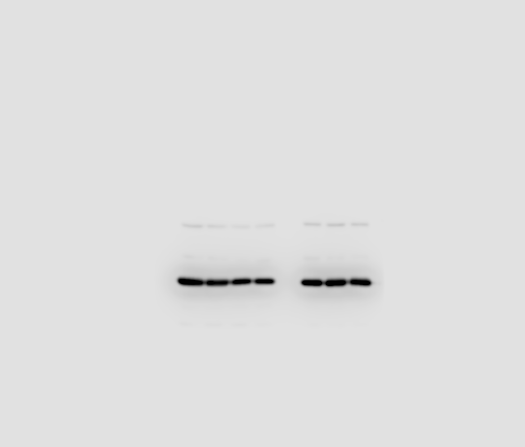

Supplement: Figure 3—source data 2. [file elife-77746-fig3-data2.zip › Fig.3/unedited/Fig.3A-GAPDH.tif]

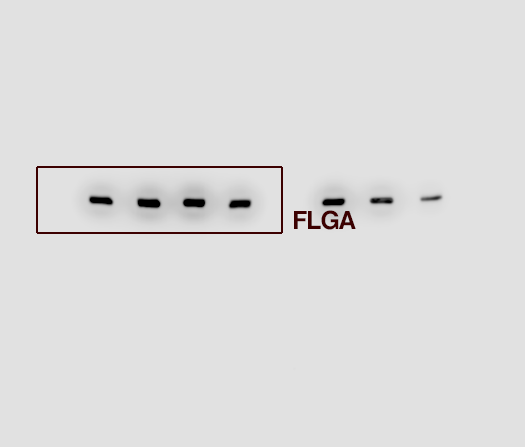

Supplement: Figure 4—source data 2. [file elife-77746-fig4-data2.zip › Fig.4/+label/Fig.4C-FLAG(input)(+label).tif]

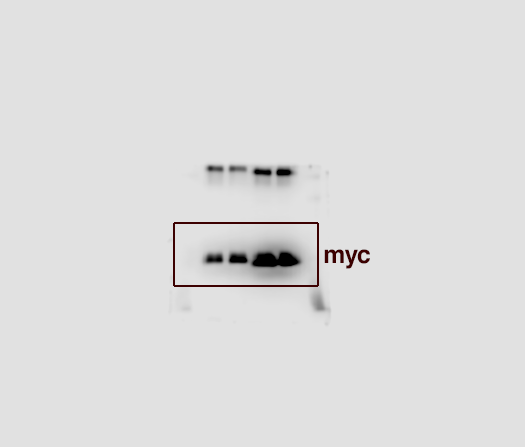

Supplement: Figure 4—source data 2. [file elife-77746-fig4-data2.zip › Fig.4/+label/Fig.4D-myc (input)(+label).tif]

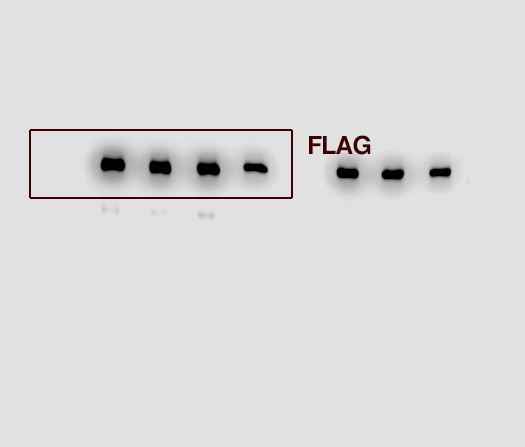

Supplement: Figure 4—source data 2. [file elife-77746-fig4-data2.zip › Fig.4/+label/Fig.4C-FLAG(IP)(+label).tif]

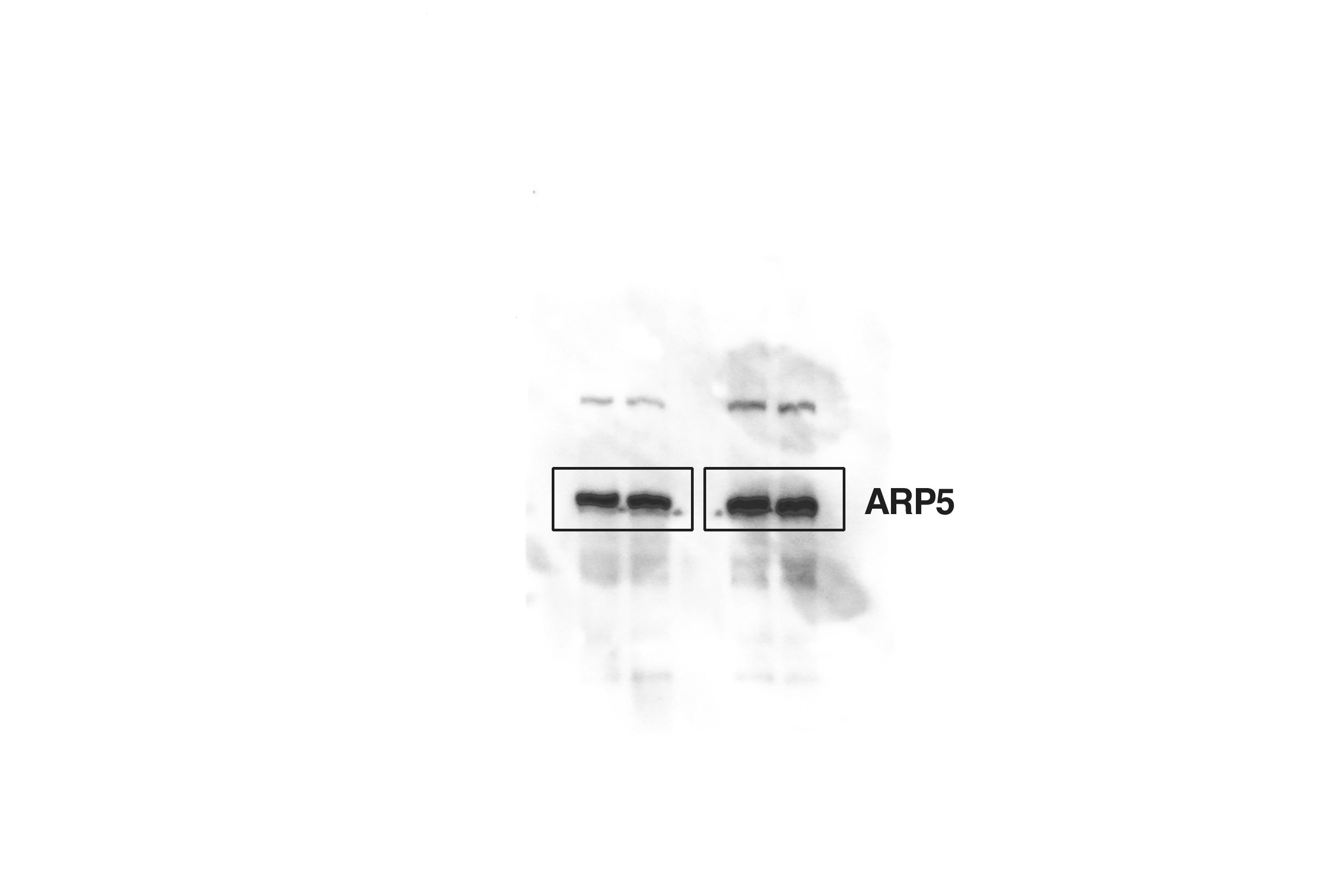

Supplement: Figure 4—source data 2. [file elife-77746-fig4-data2.zip › Fig.4/+label/Fig.4B-Arp5(input)(+label).tif]

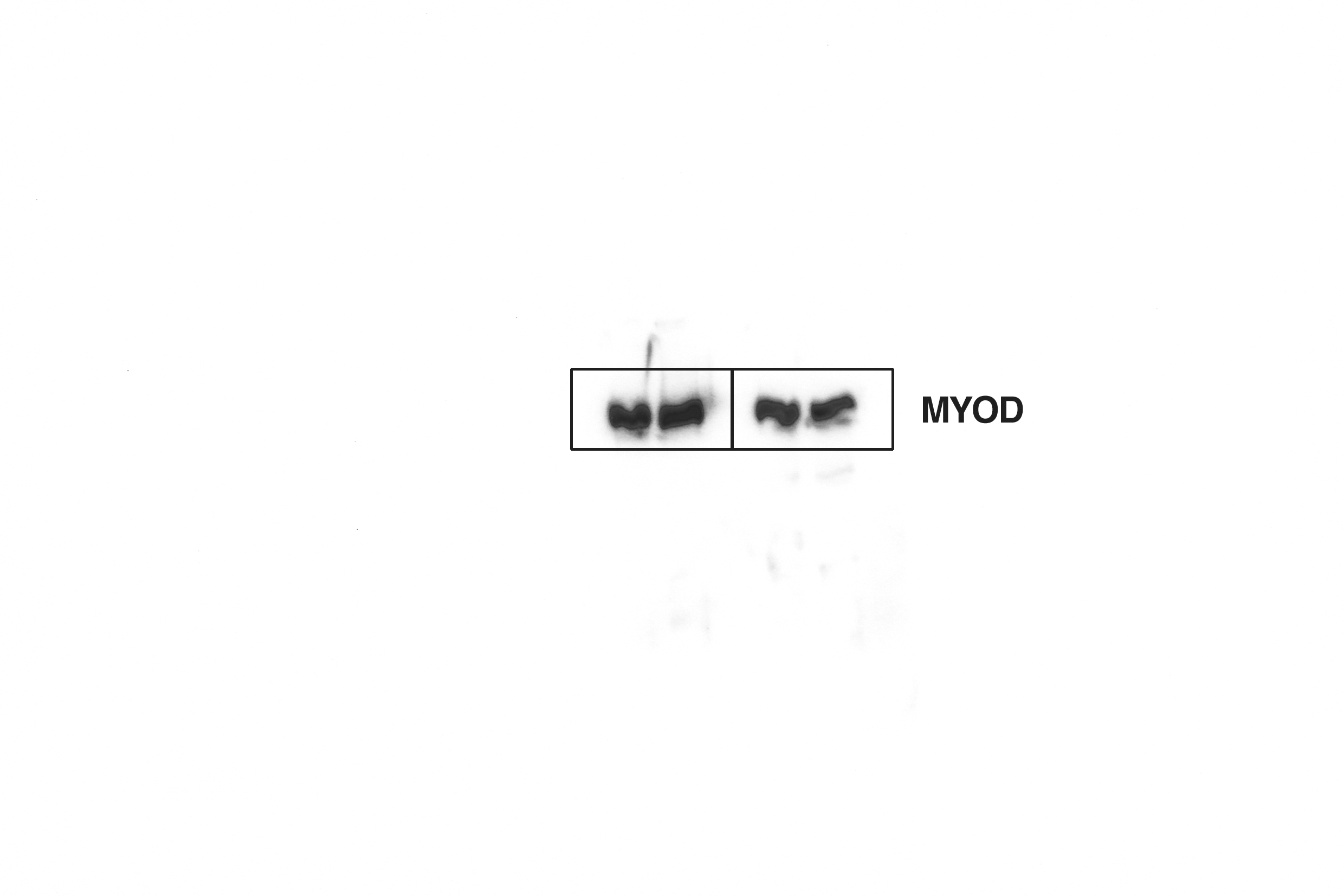

Supplement: Figure 4—source data 2. [file elife-77746-fig4-data2.zip › Fig.4/+label/Fig.4B-MyoD(input)(+label).tif]

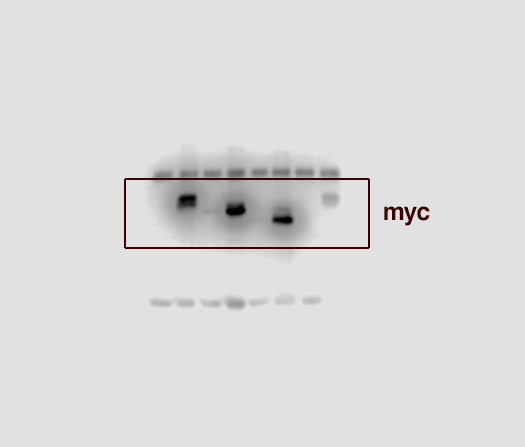

Supplement: Figure 4—source data 2. [file elife-77746-fig4-data2.zip › Fig.4/+label/Fig.4C-Myc(IP)(+label).tif]

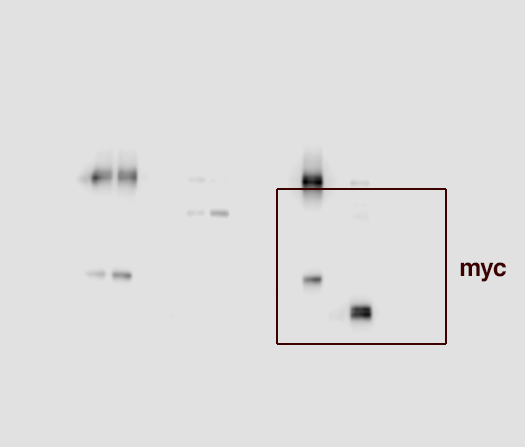

Supplement: Figure 4—source data 2. [file elife-77746-fig4-data2.zip › Fig.4/+label/Fig.4A-myc(IP)(+label).tif]

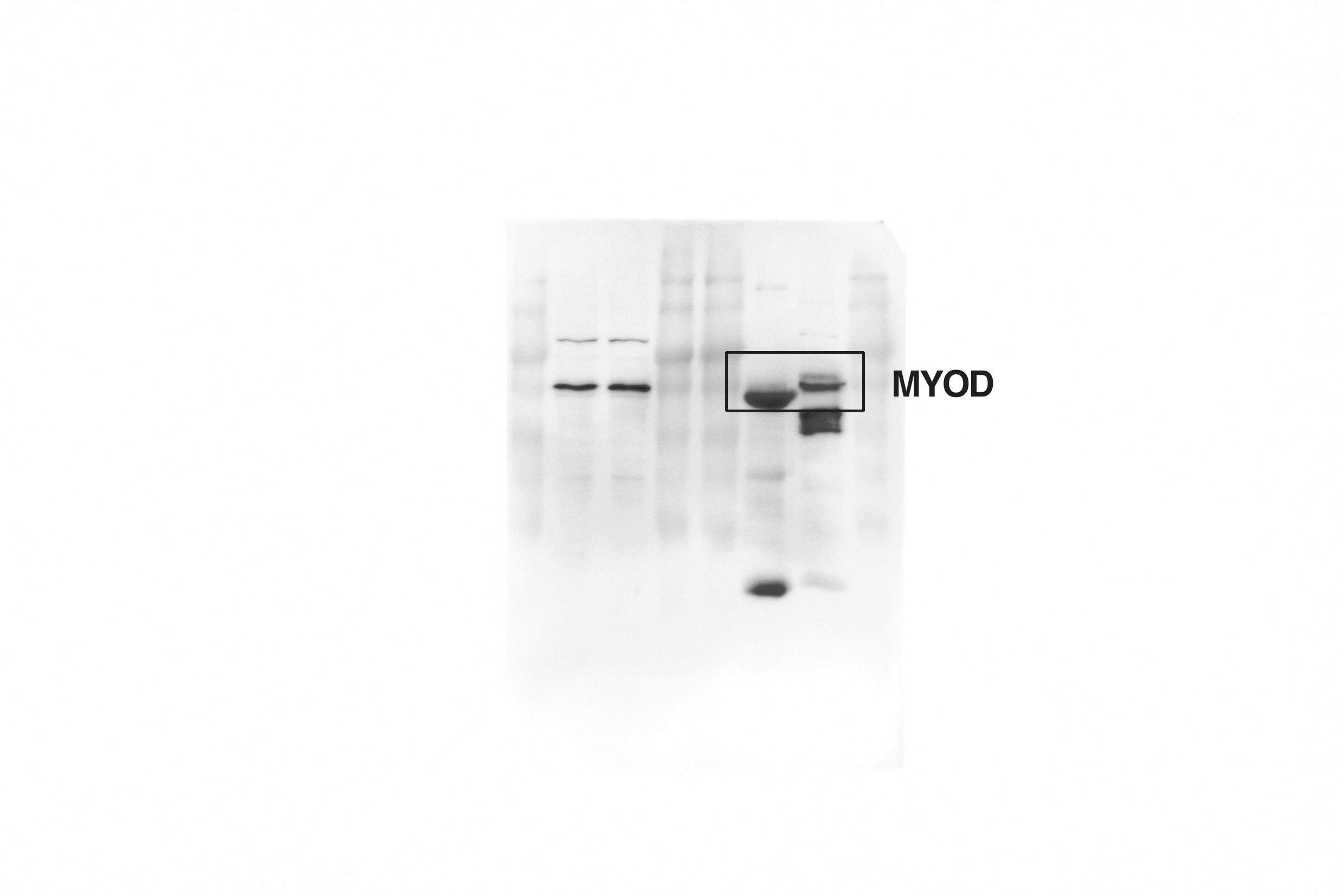

Supplement: Figure 4—source data 2. [file elife-77746-fig4-data2.zip › Fig.4/+label/Fig.4B-MyoD(IP)(+label).tif]

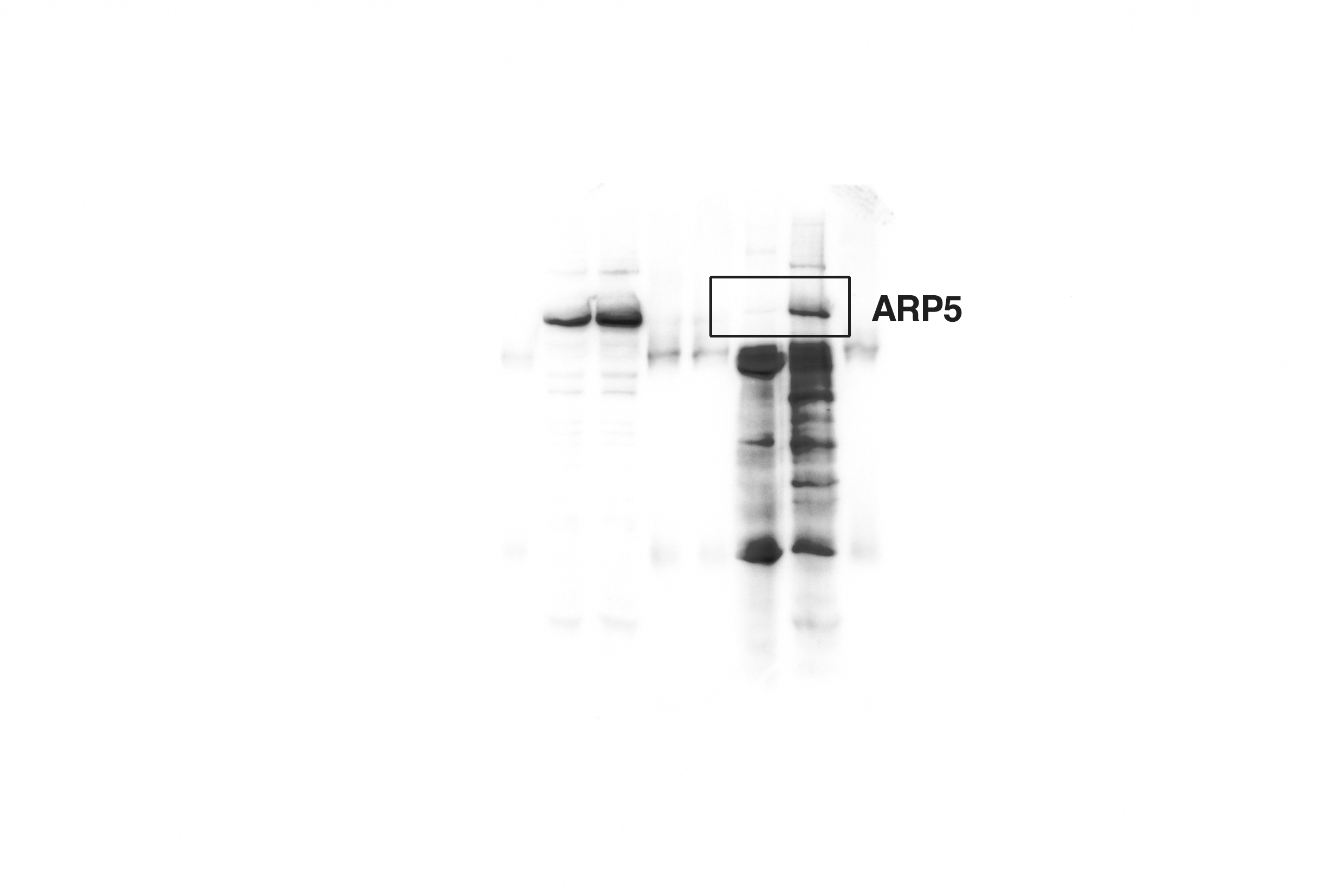

Supplement: Figure 4—source data 2. [file elife-77746-fig4-data2.zip › Fig.4/+label/Fig.4B-Arp5(pulldown)(+label).tif]

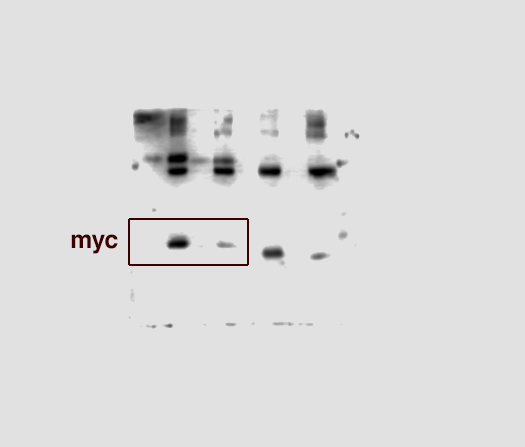

Supplement: Figure 4—source data 2. [file elife-77746-fig4-data2.zip › Fig.4/+label/Fig.4D-myc(IP)(+label).tif]

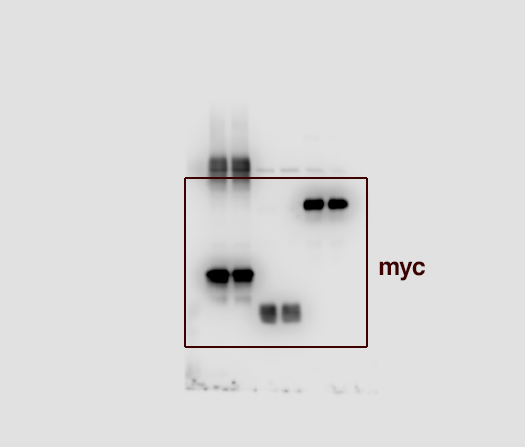

Supplement: Figure 4—source data 2. [file elife-77746-fig4-data2.zip › Fig.4/+label/Fig.4A-myc(input)(+label).tif]

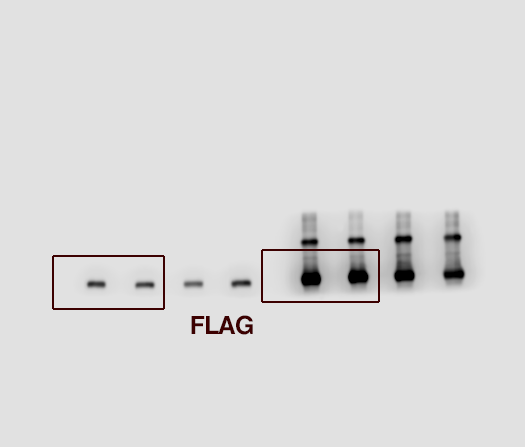

Supplement: Figure 4—source data 2. [file elife-77746-fig4-data2.zip › Fig.4/+label/Fig.4D-FLAG(+label).tif]

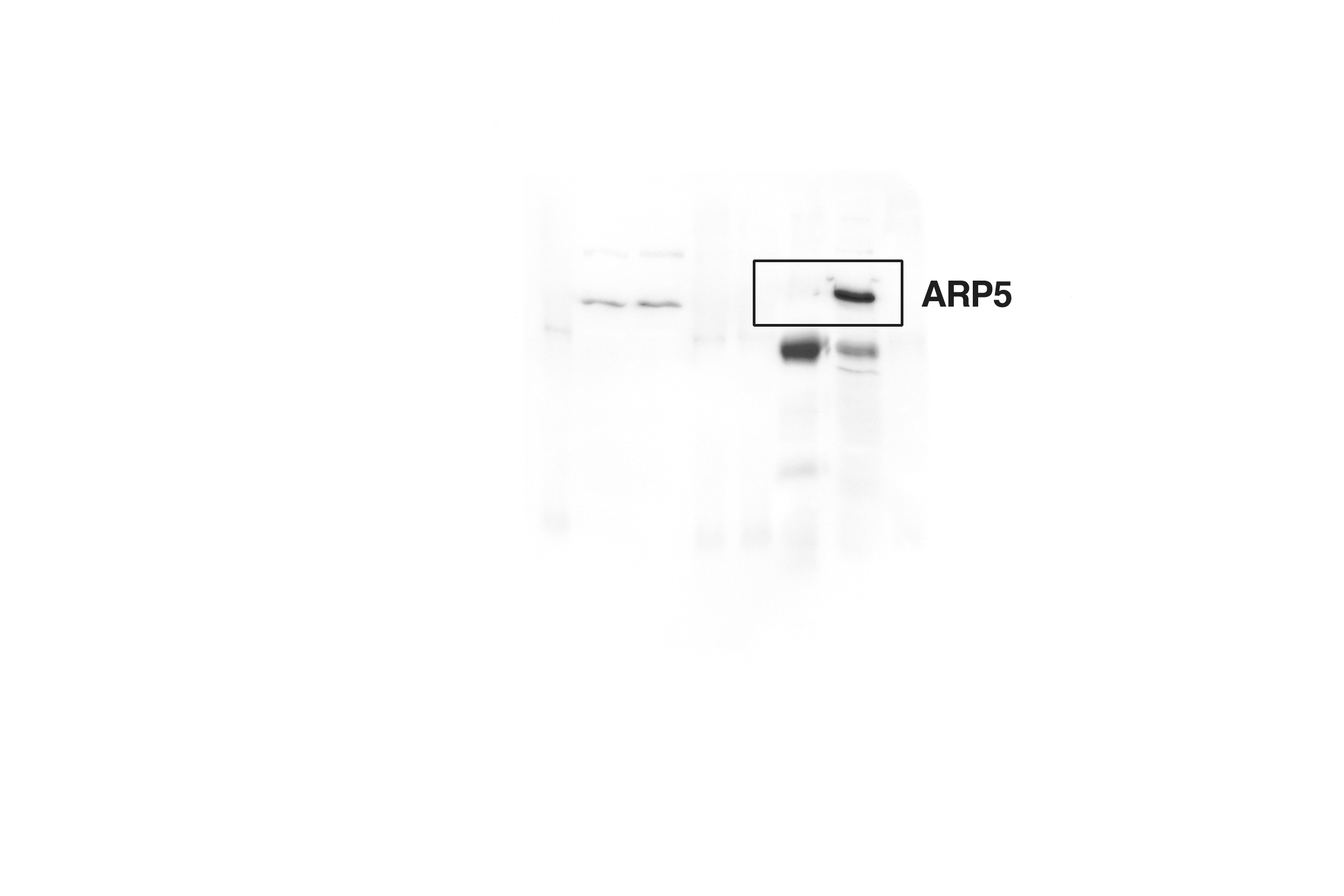

Supplement: Figure 4—source data 2. [file elife-77746-fig4-data2.zip › Fig.4/+label/Fig.4B-Arp5(IP)(+label).tif]

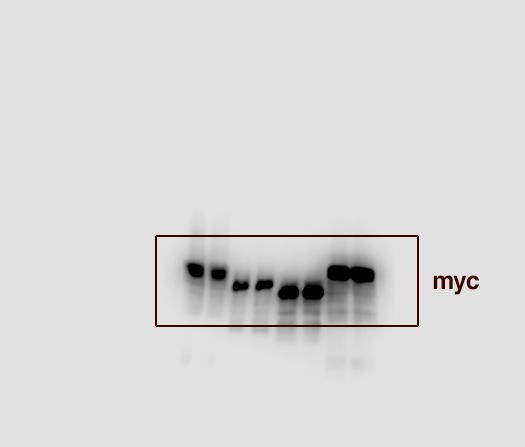

Supplement: Figure 4—source data 2. [file elife-77746-fig4-data2.zip › Fig.4/+label/Fig.4C-myc(input)(+label).tif]

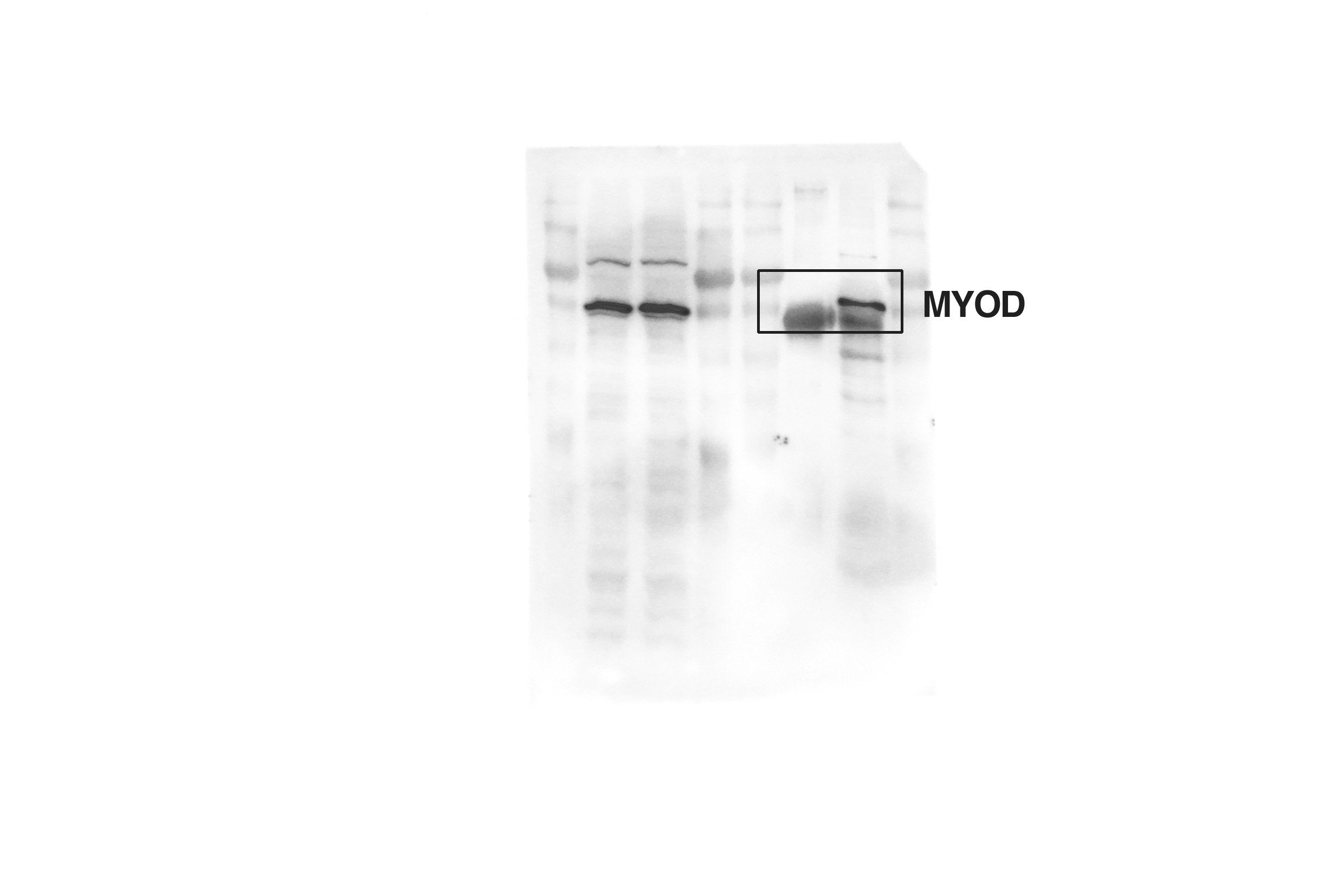

Supplement: Figure 4—source data 2. [file elife-77746-fig4-data2.zip › Fig.4/+label/Fig.4B-MyoD(pulldown)(+label).tif]

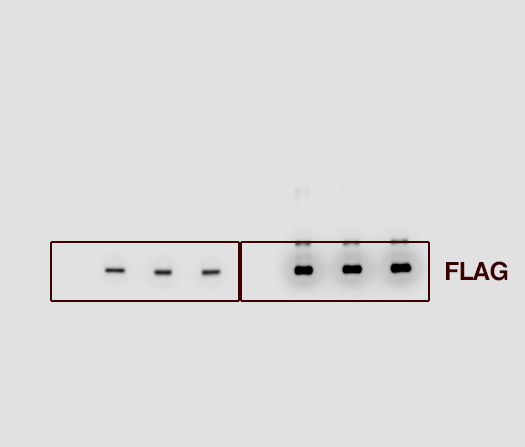

Supplement: Figure 4—source data 2. [file elife-77746-fig4-data2.zip › Fig.4/+label/Fig.4A-FLAG(+label).tif]

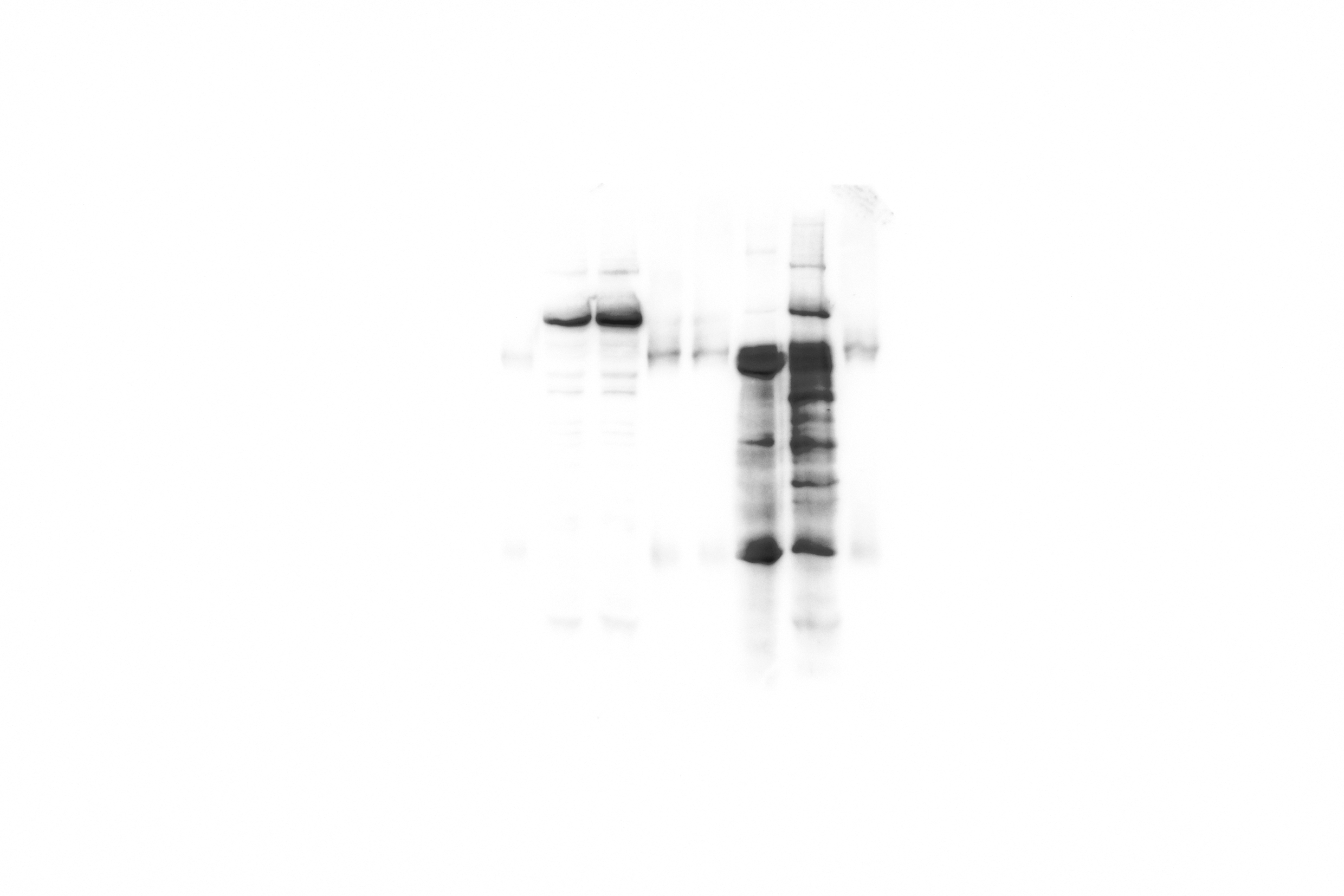

Supplement: Figure 4—source data 2. [file elife-77746-fig4-data2.zip › Fig.4/unedited/Fig.4B-Arp5(pulldown).tif]

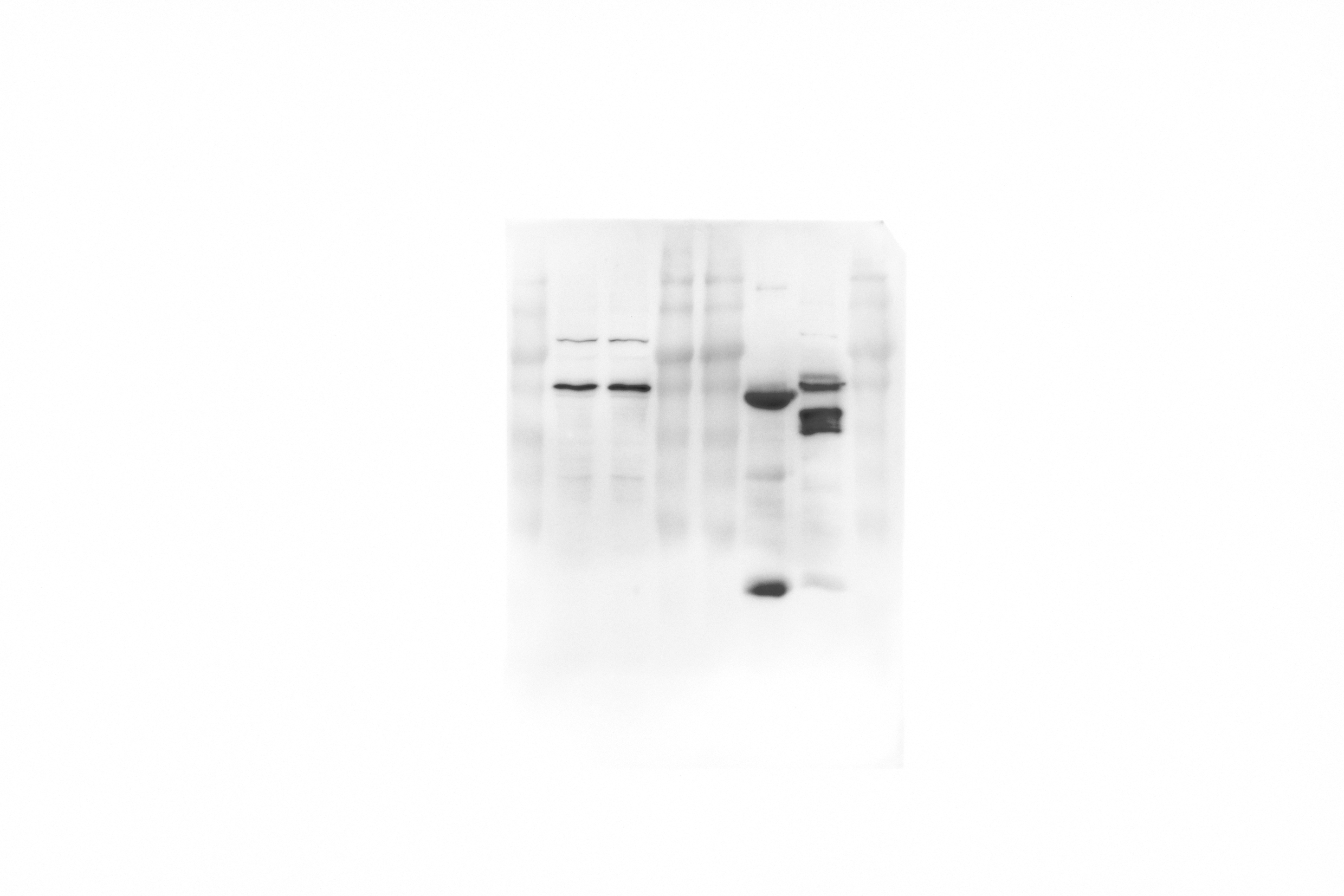

Supplement: Figure 4—source data 2. [file elife-77746-fig4-data2.zip › Fig.4/unedited/Fig.4B-MyoD(IP).tif]

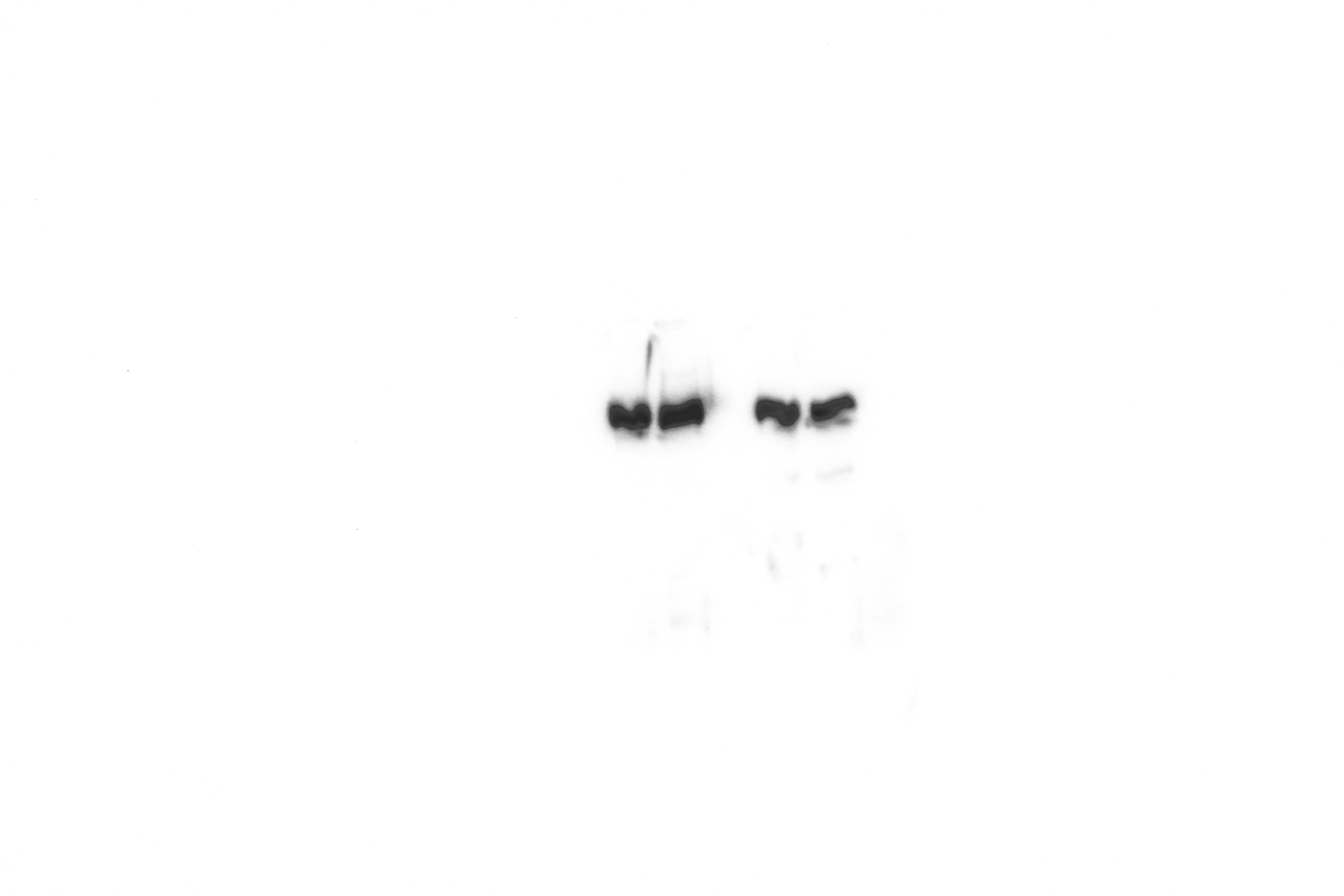

Supplement: Figure 4—source data 2. [file elife-77746-fig4-data2.zip › Fig.4/unedited/Fig.4B-MyoD(input).tif]

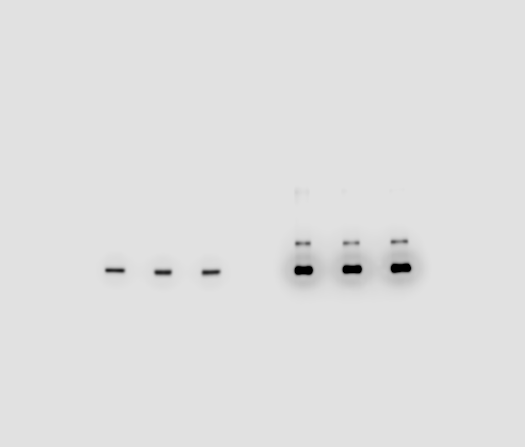

Supplement: Figure 4—source data 2. [file elife-77746-fig4-data2.zip › Fig.4/unedited/Fig.4A-FLAG.tif]

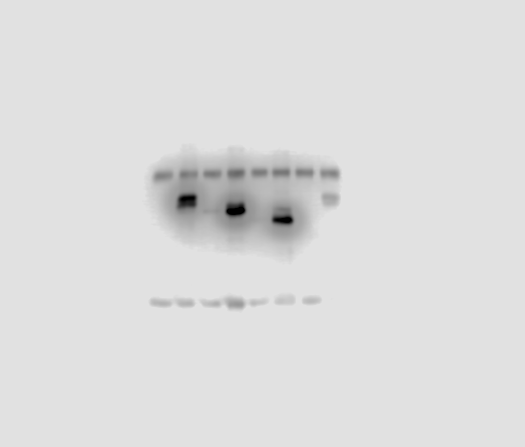

Supplement: Figure 4—source data 2. [file elife-77746-fig4-data2.zip › Fig.4/unedited/Fig.4C-Myc(IP).tif]

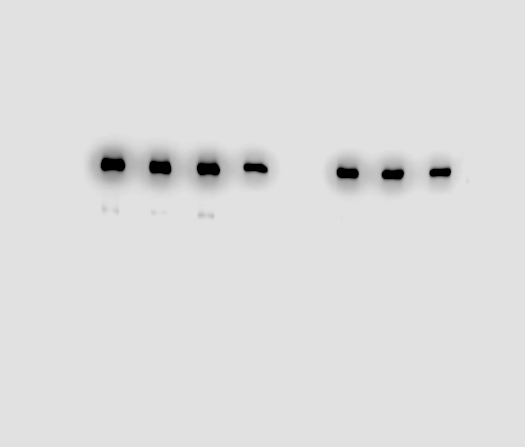

Supplement: Figure 4—source data 2. [file elife-77746-fig4-data2.zip › Fig.4/unedited/Fig.4C-FLAG(IP).tif]

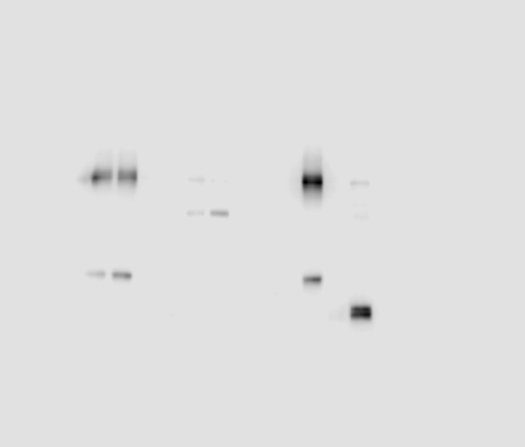

Supplement: Figure 4—source data 2. [file elife-77746-fig4-data2.zip › Fig.4/unedited/Fig.4A-myc(IP).tif]

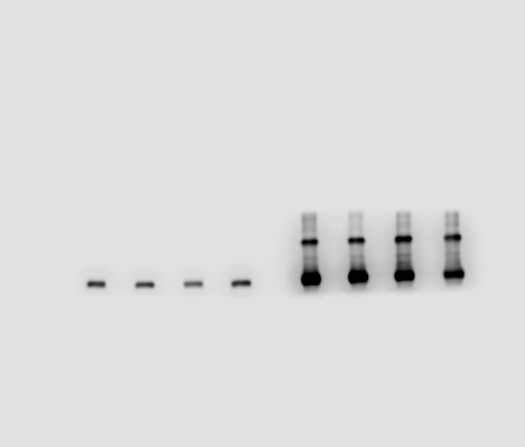

Supplement: Figure 4—source data 2. [file elife-77746-fig4-data2.zip › Fig.4/unedited/Fig.4D-FLAG.tif]

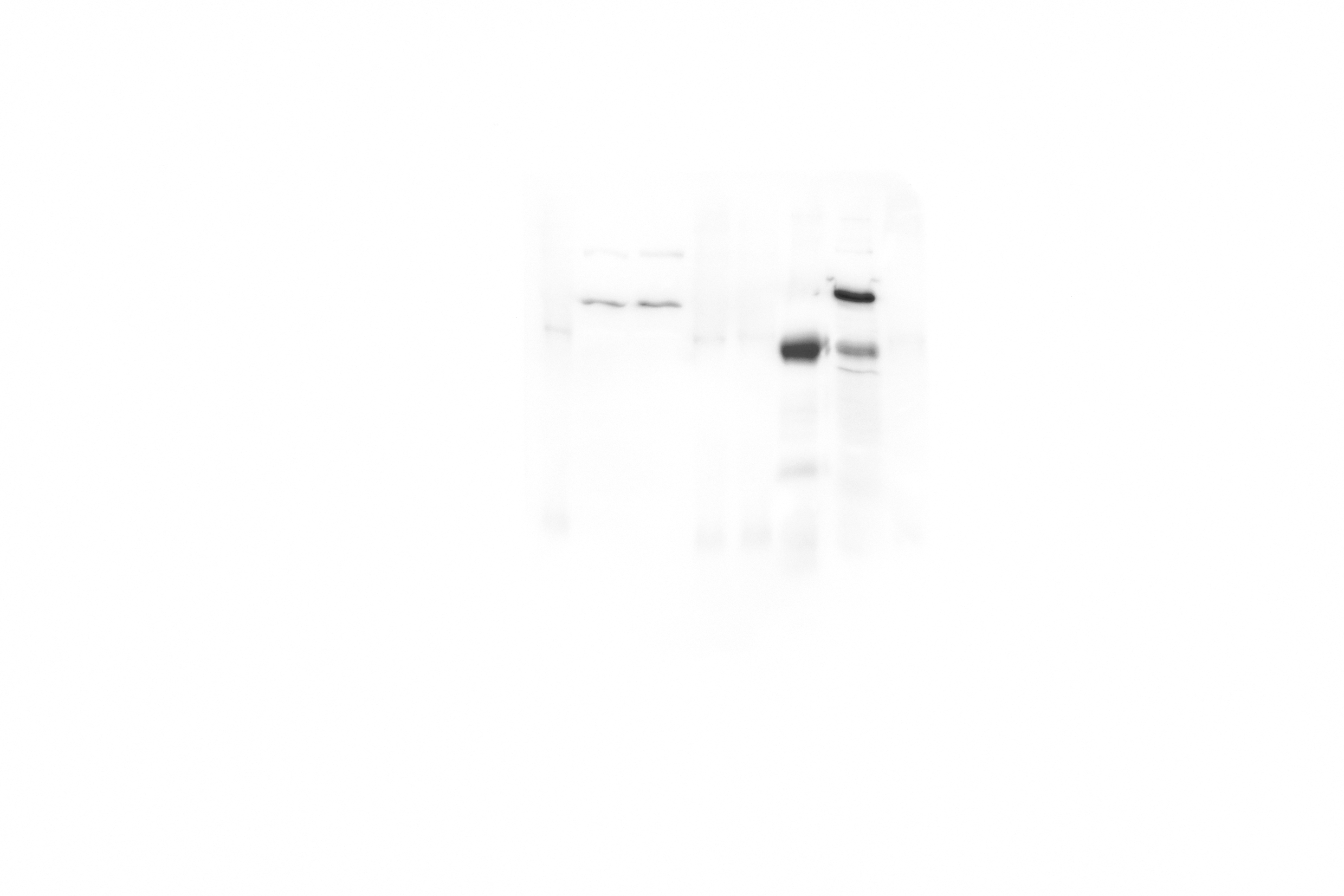

Supplement: Figure 4—source data 2. [file elife-77746-fig4-data2.zip › Fig.4/unedited/Fig.4B-Arp5(IP).tif]

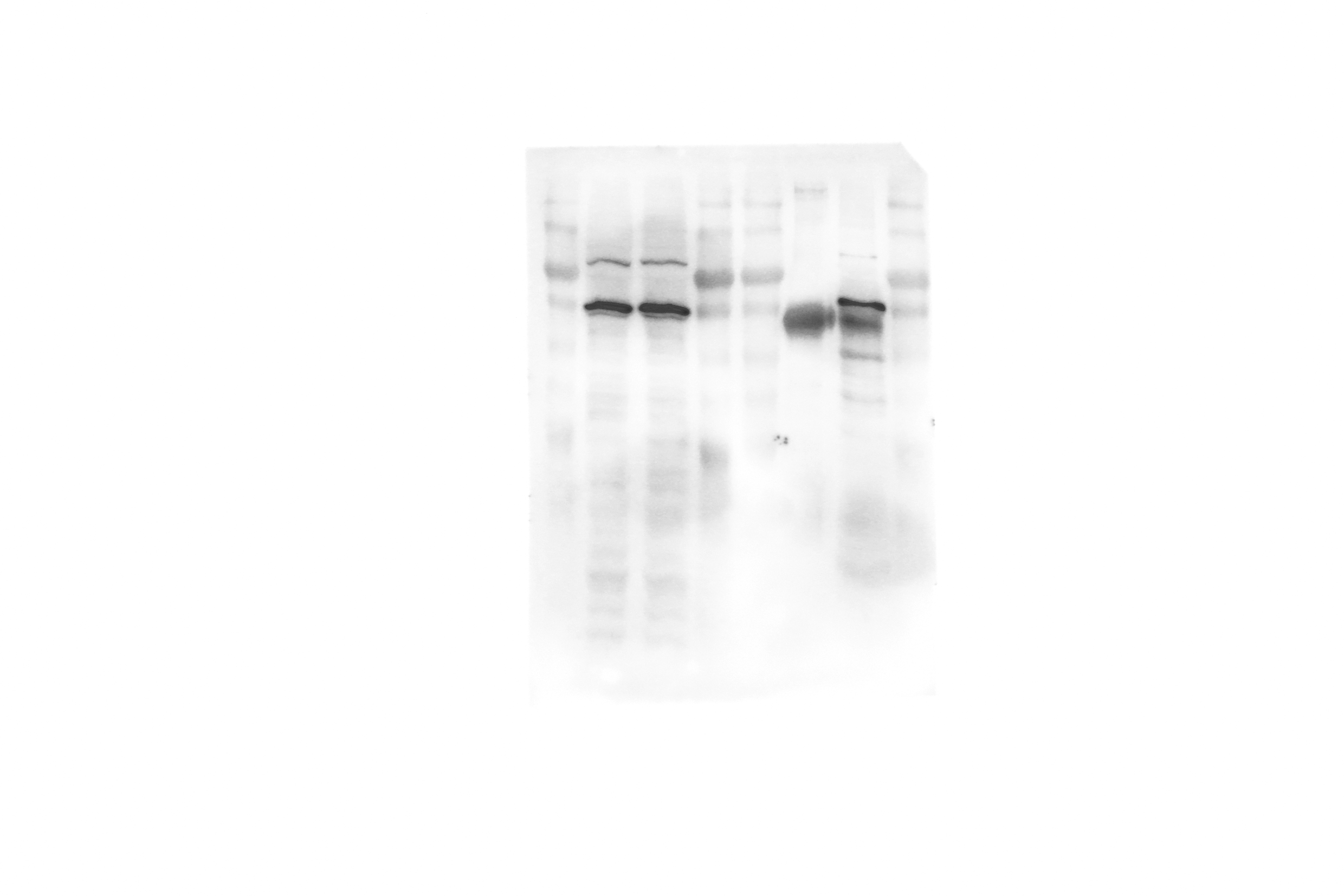

Supplement: Figure 4—source data 2. [file elife-77746-fig4-data2.zip › Fig.4/unedited/Fig.4B-MyoD(pulldown).tif]

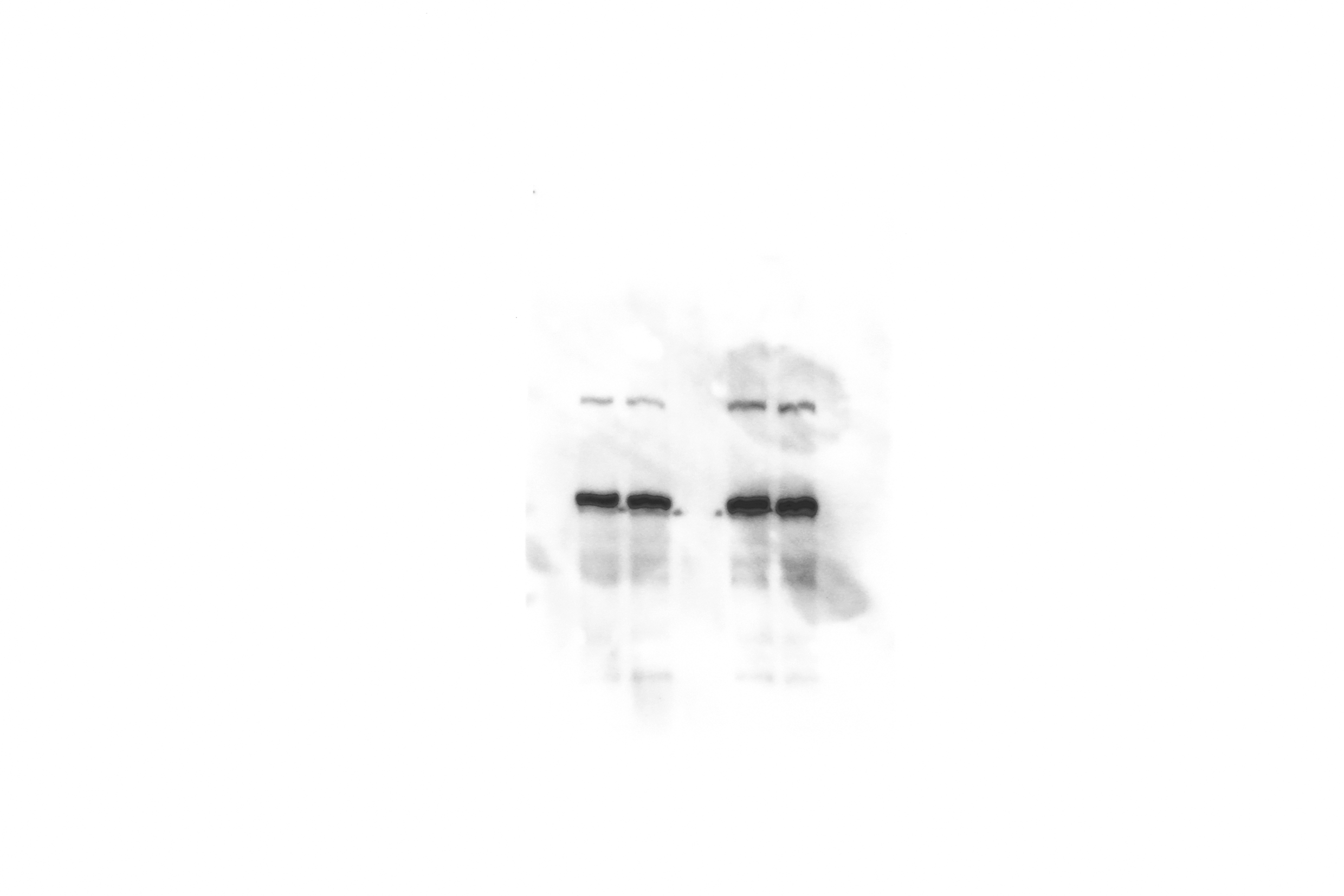

Supplement: Figure 4—source data 2. [file elife-77746-fig4-data2.zip › Fig.4/unedited/Fig.4B-Arp5(input).tif]

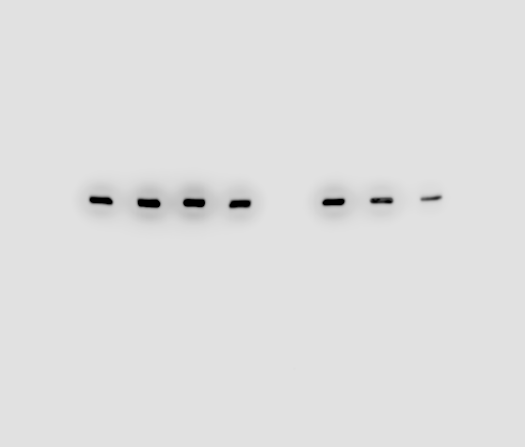

Supplement: Figure 4—source data 2. [file elife-77746-fig4-data2.zip › Fig.4/unedited/Fig.4C-FLAG(input).tif]

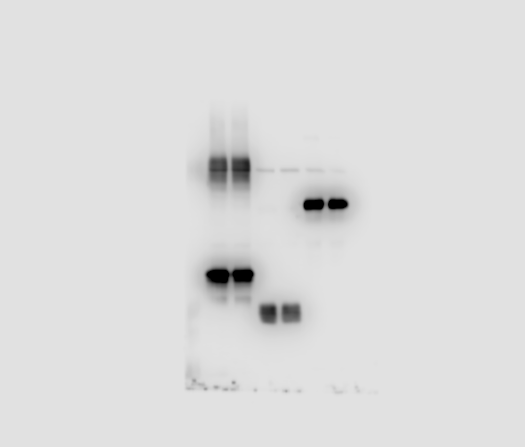

Supplement: Figure 4—source data 2. [file elife-77746-fig4-data2.zip › Fig.4/unedited/Fig.4A-myc(input).tif]

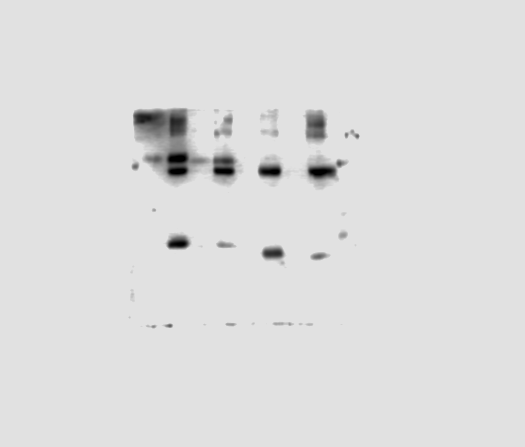

Supplement: Figure 4—source data 2. [file elife-77746-fig4-data2.zip › Fig.4/unedited/Fig.4D-myc(IP).tif]

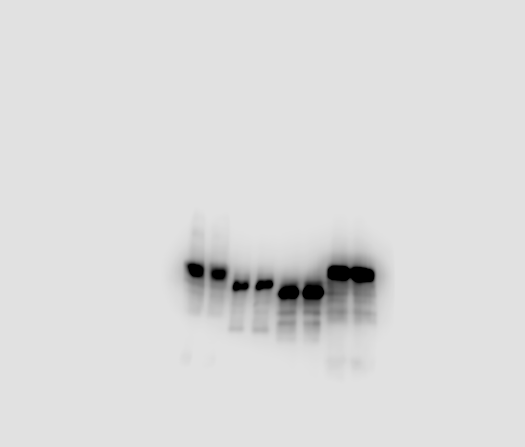

Supplement: Figure 4—source data 2. [file elife-77746-fig4-data2.zip › Fig.4/unedited/Fig.4C-myc(input).tif]

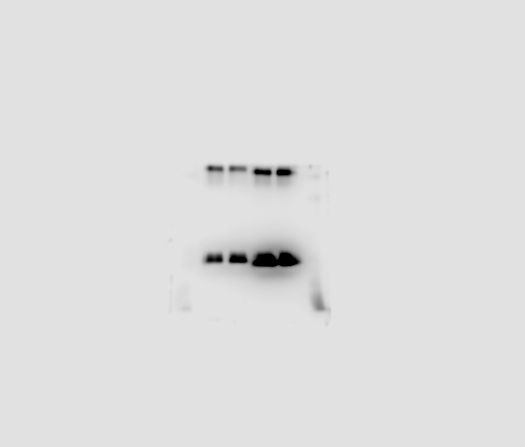

Supplement: Figure 4—source data 2. [file elife-77746-fig4-data2.zip › Fig.4/unedited/Fig.4D-myc (input).tif]

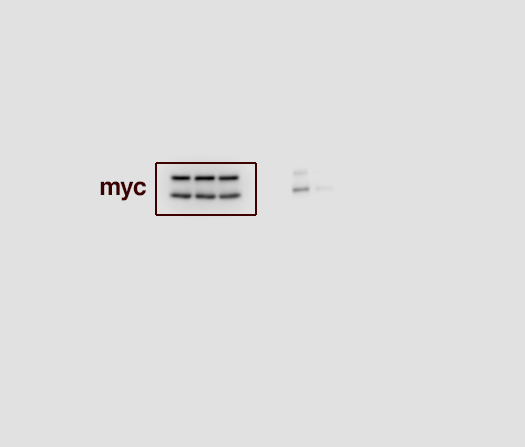

Supplement: Figure 5—source data 2. [file elife-77746-fig5-data2.zip › Fig.5/+label/Fig.5B-myc(input)(+label).tif]

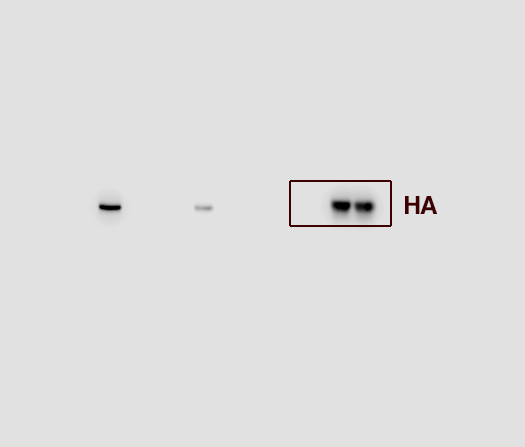

Supplement: Figure 5—source data 2. [file elife-77746-fig5-data2.zip › Fig.5/+label/Fig.5B-FLAG&HA(IP)(+label).tif]

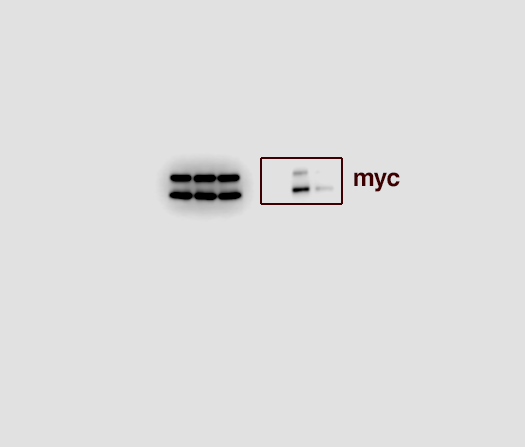

Supplement: Figure 5—source data 2. [file elife-77746-fig5-data2.zip › Fig.5/+label/Fig.5B-myc(IP)(+label).tif]

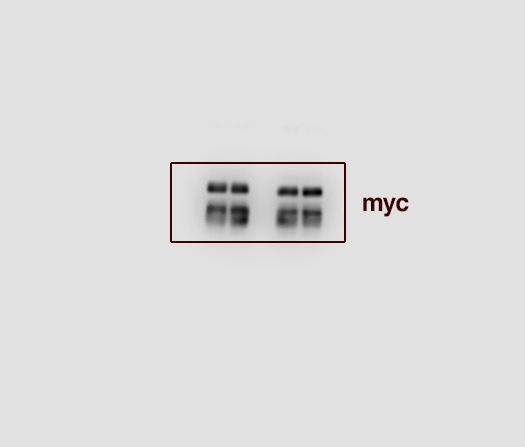

Supplement: Figure 5—source data 2. [file elife-77746-fig5-data2.zip › Fig.5/+label/Fig.5E-myc(input)(+label).tif]

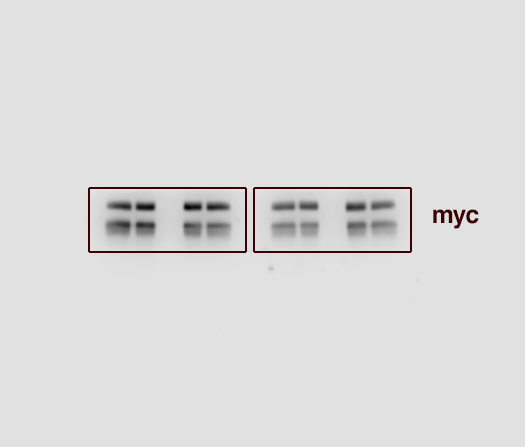

Supplement: Figure 5—source data 2. [file elife-77746-fig5-data2.zip › Fig.5/+label/Fig.5F-myc(input)(+label).tif]

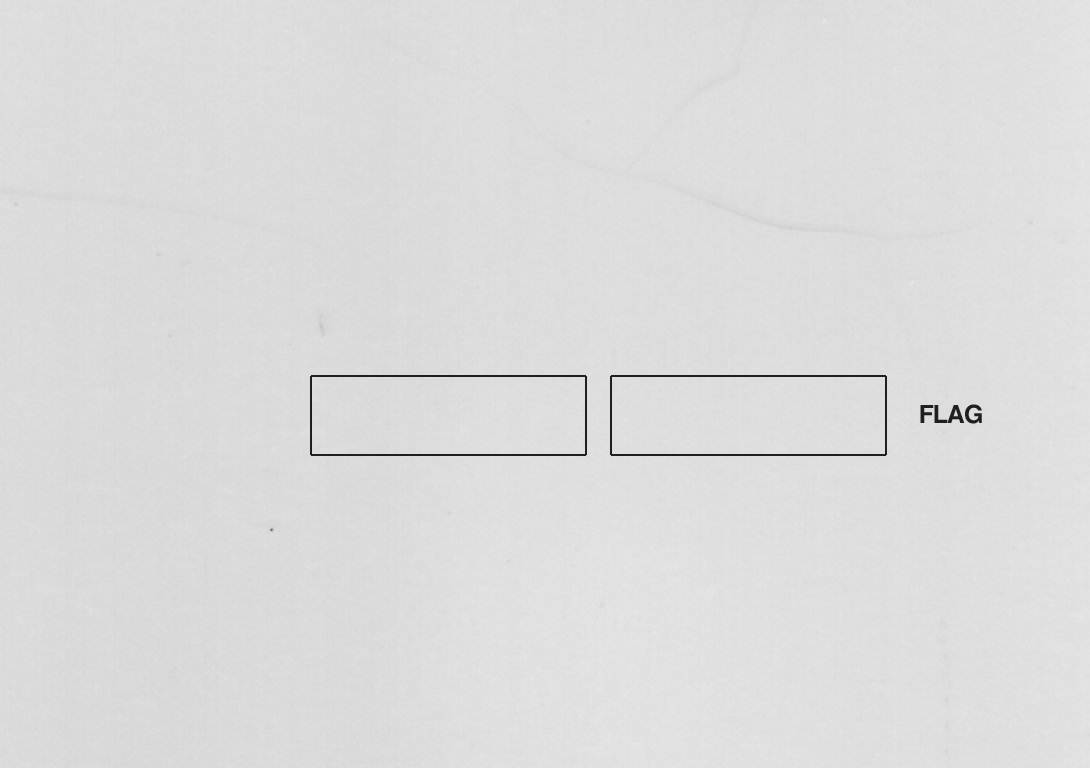

Supplement: Figure 5—source data 2. [file elife-77746-fig5-data2.zip › Fig.5/+label/FIg.5F-FLAG(pulldown)(+label).tif]

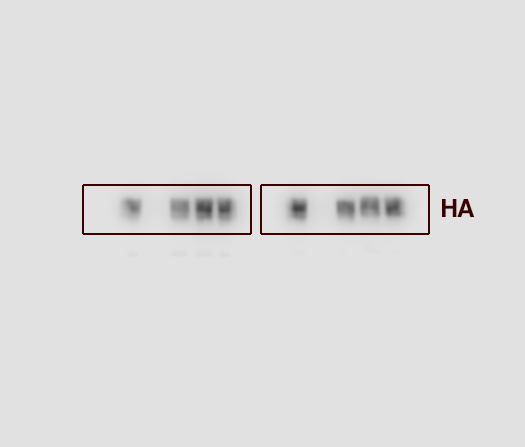

Supplement: Figure 5—source data 2. [file elife-77746-fig5-data2.zip › Fig.5/+label/Fig.5F-HA(Myod input)(+label).tif]

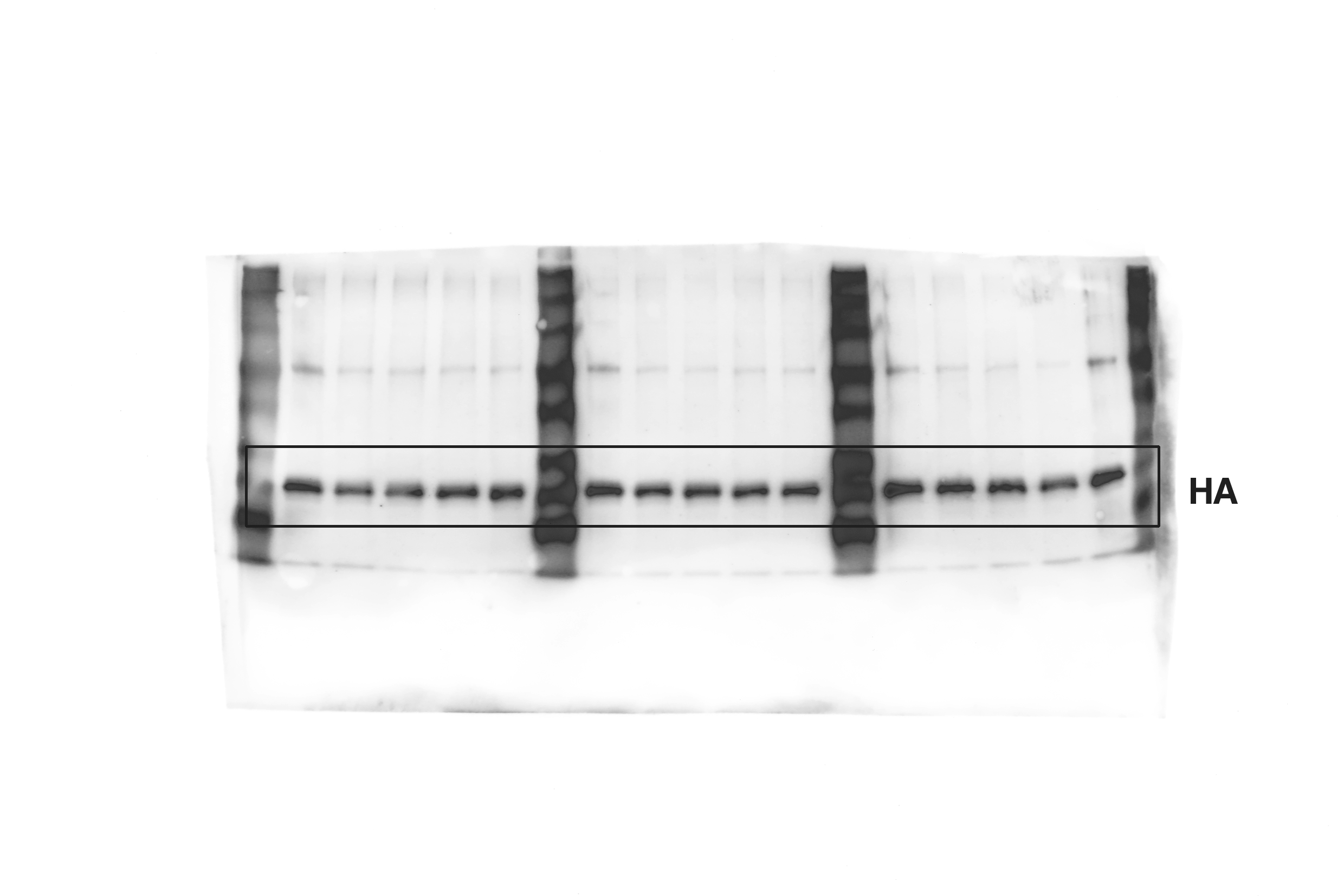

Supplement: Figure 5—source data 2. [file elife-77746-fig5-data2.zip › Fig.5/+label/Fig.5A-HA(+label).tif]

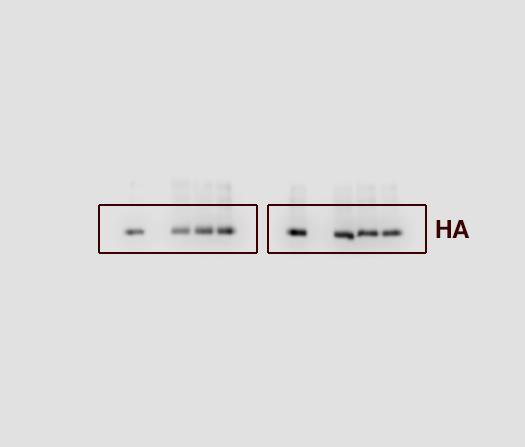

Supplement: Figure 5—source data 2. [file elife-77746-fig5-data2.zip › Fig.5/+label/Fig.5F-HA(E47-input)(+label).tif]

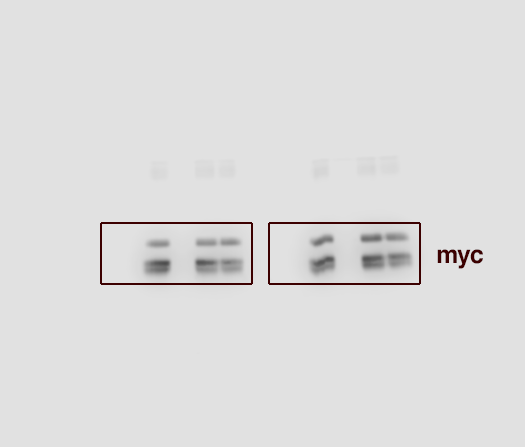

Supplement: Figure 5—source data 2. [file elife-77746-fig5-data2.zip › Fig.5/+label/Fig.5F-myc(pulldown)(+label).tif]

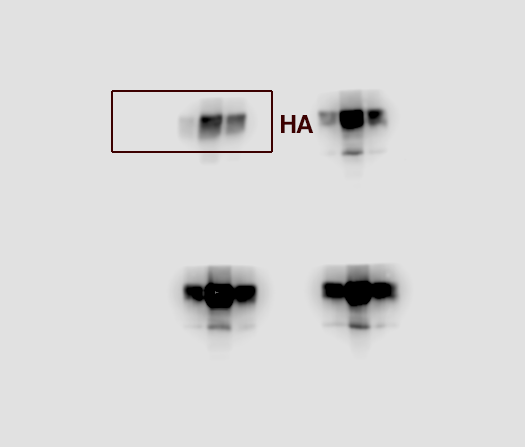

Supplement: Figure 5—source data 2. [file elife-77746-fig5-data2.zip › Fig.5/+label/Fig.5F-HA(myoD PME1-pulldown)(+label).tif]

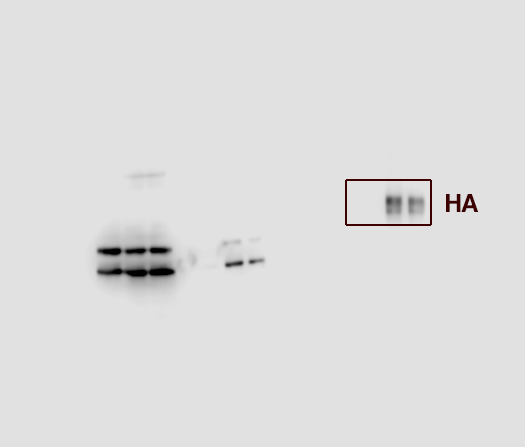

Supplement: Figure 5—source data 2. [file elife-77746-fig5-data2.zip › Fig.5/+label/Fig.5B-HA(input)(+label).tif]

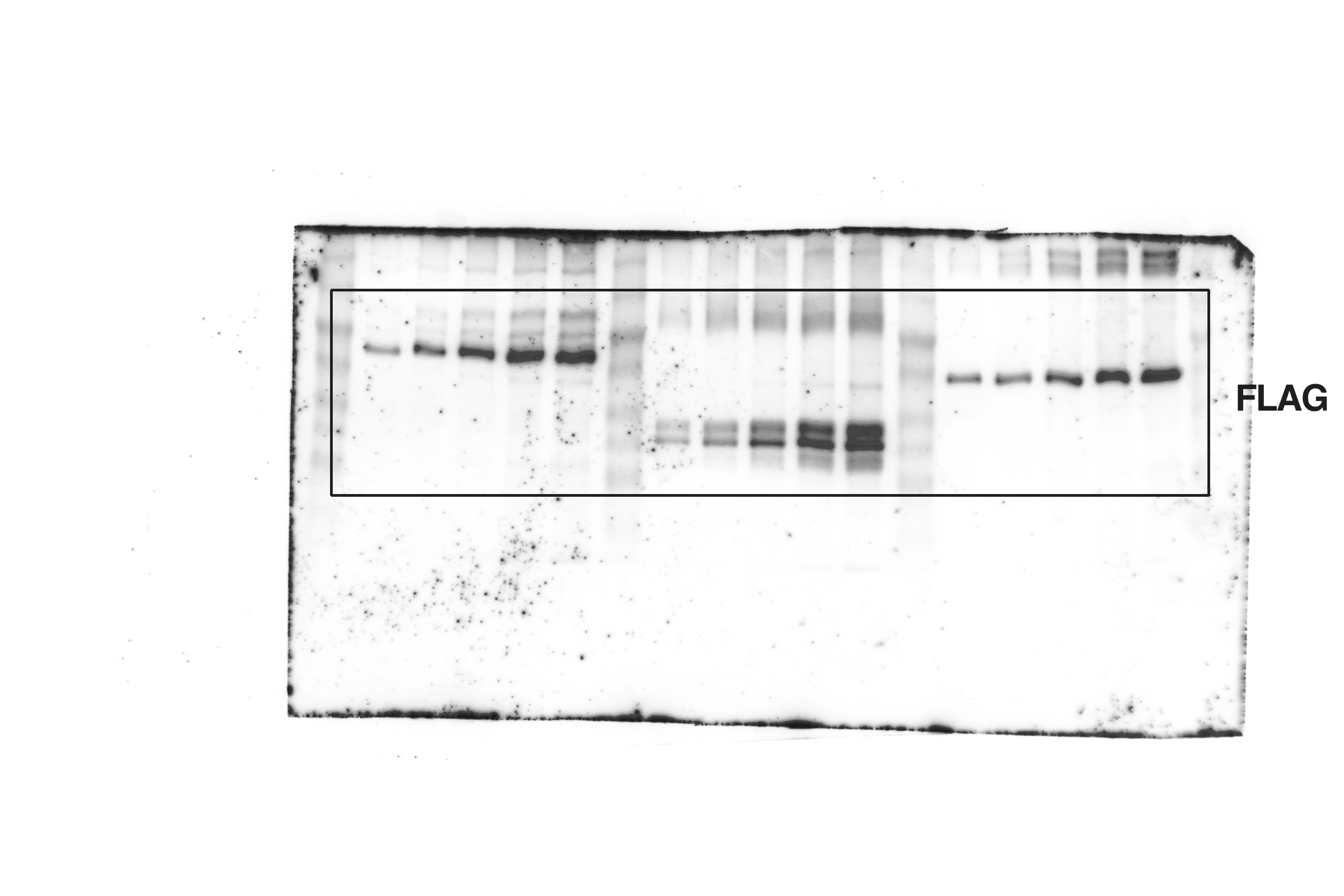

Supplement: Figure 5—source data 2. [file elife-77746-fig5-data2.zip › Fig.5/+label/Fig.5A-FLAG(input)(+label).tif]

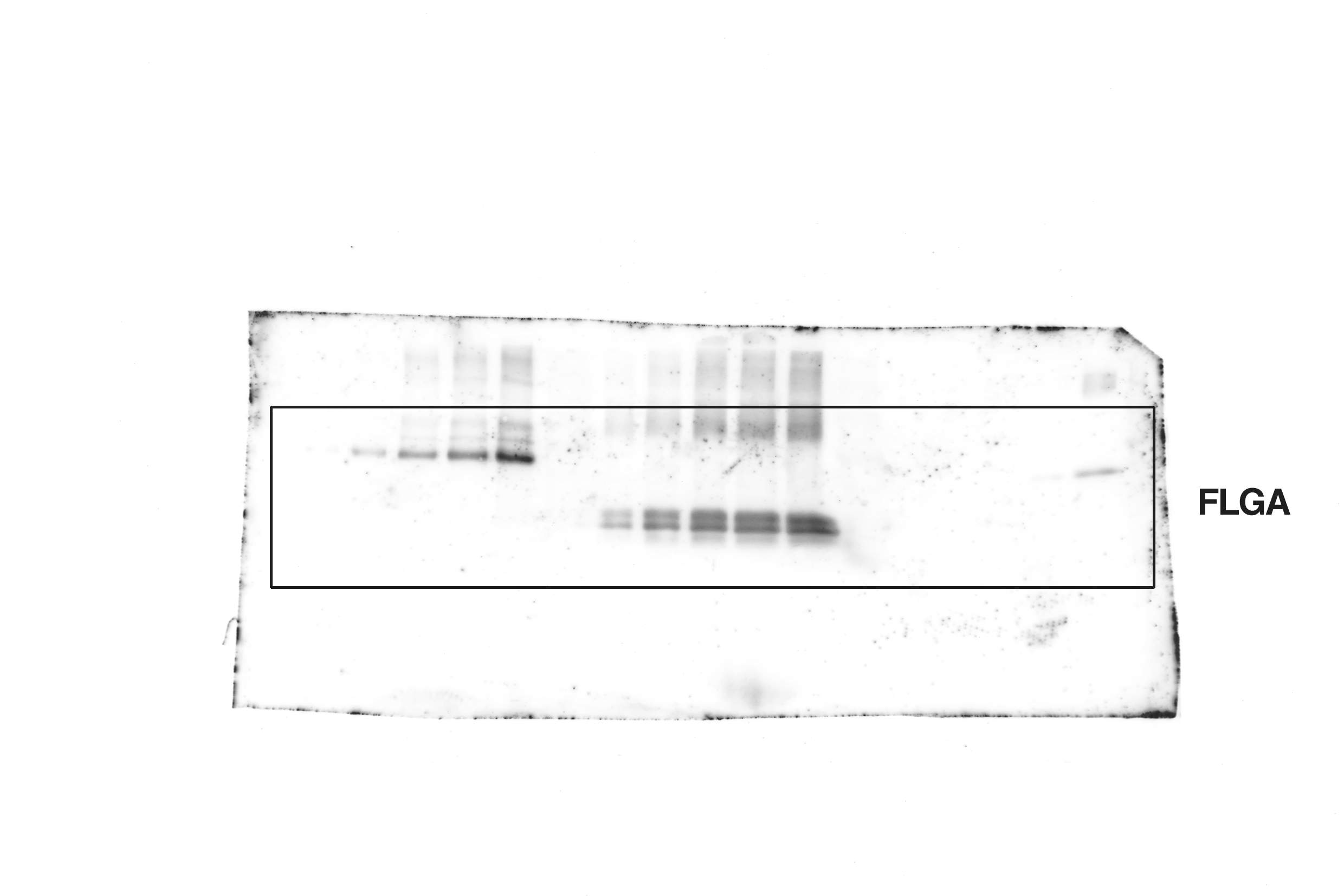

Supplement: Figure 5—source data 2. [file elife-77746-fig5-data2.zip › Fig.5/+label/Fig.5A-FLAG(elute)(+label).tif]

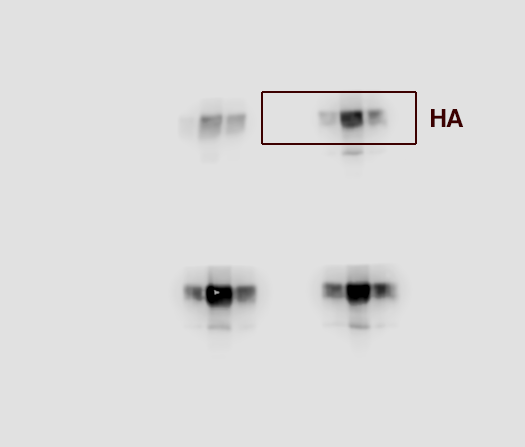

Supplement: Figure 5—source data 2. [file elife-77746-fig5-data2.zip › Fig.5/+label/Fig.5F-HA(MyoD PME2-pulldown)(+label).tif]

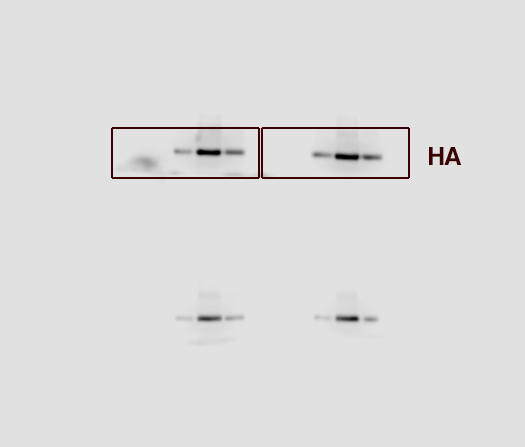

Supplement: Figure 5—source data 2. [file elife-77746-fig5-data2.zip › Fig.5/+label/Fig.5F-HA(E47-pulldown)(+label).tif]

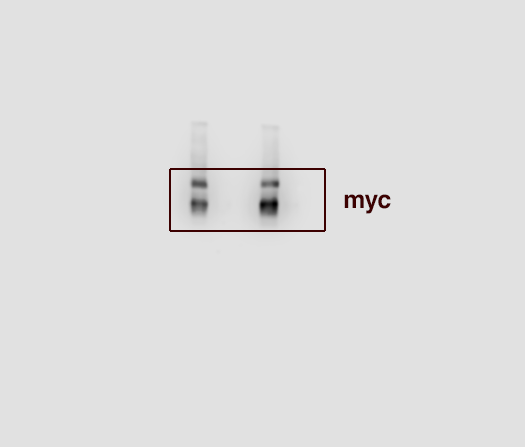

Supplement: Figure 5—source data 2. [file elife-77746-fig5-data2.zip › Fig.5/+label/Fig.5E-myc(pulldown)(+label).tif]

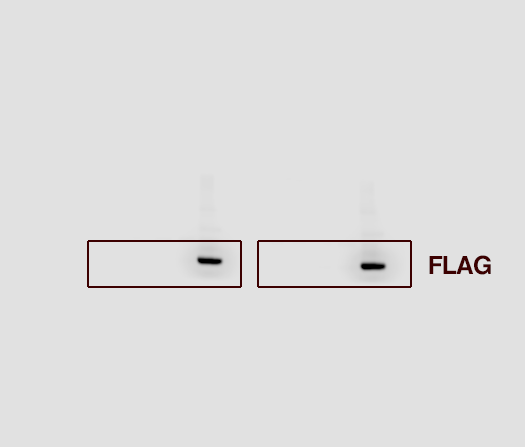

Supplement: Figure 5—source data 2. [file elife-77746-fig5-data2.zip › Fig.5/+label/Fig.5F-FLAG(input)(+label).tif]

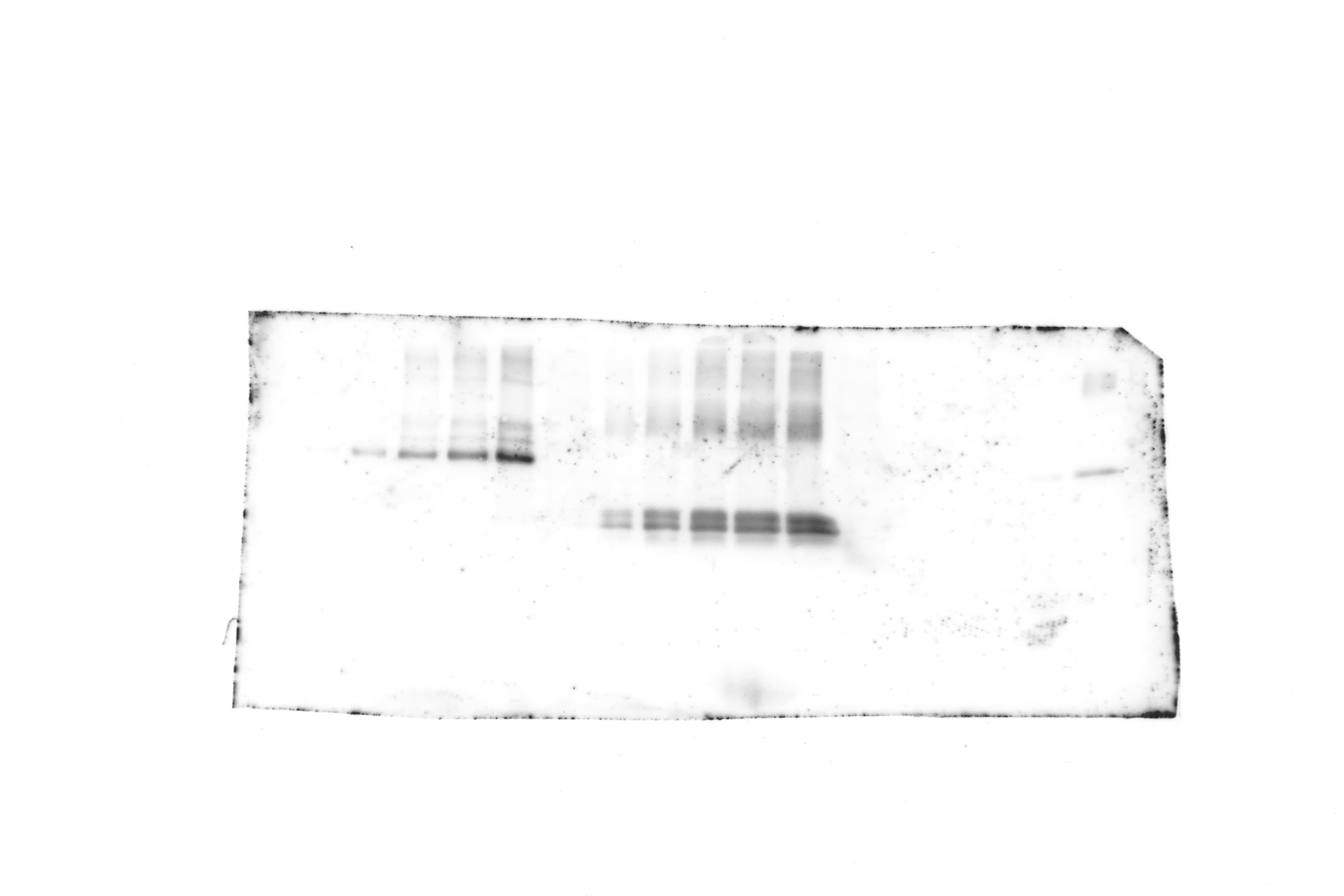

Supplement: Figure 5—source data 2. [file elife-77746-fig5-data2.zip › Fig.5/unedited/Fig.5A-FLAG(elute).tif]

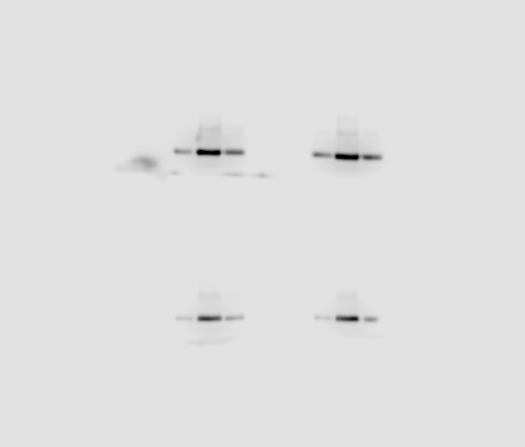

Supplement: Figure 5—source data 2. [file elife-77746-fig5-data2.zip › Fig.5/unedited/Fig.5F-HA(E47-pulldown).tif]

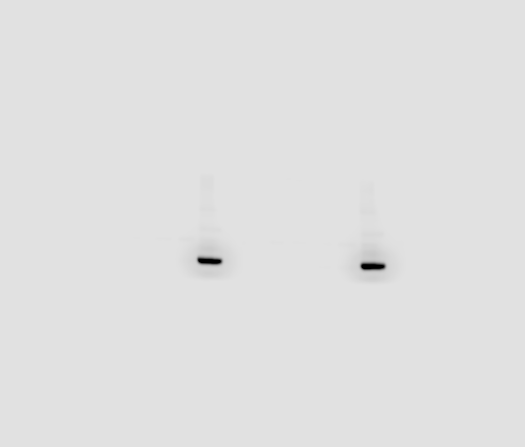

Supplement: Figure 5—source data 2. [file elife-77746-fig5-data2.zip › Fig.5/unedited/Fig.5F-FLAG(input).tif]

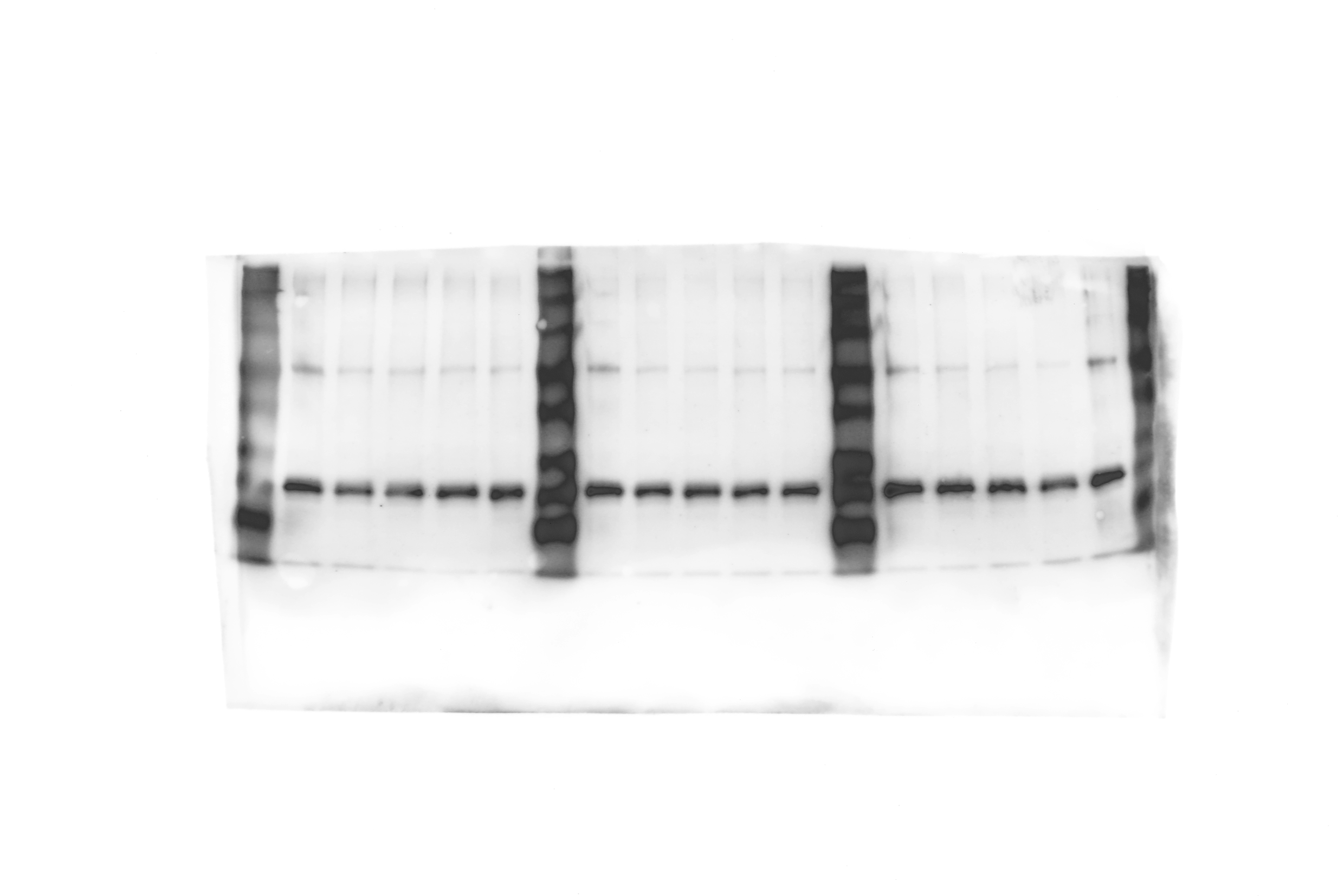

Supplement: Figure 5—source data 2. [file elife-77746-fig5-data2.zip › Fig.5/unedited/Fig.5A-HA.tif]

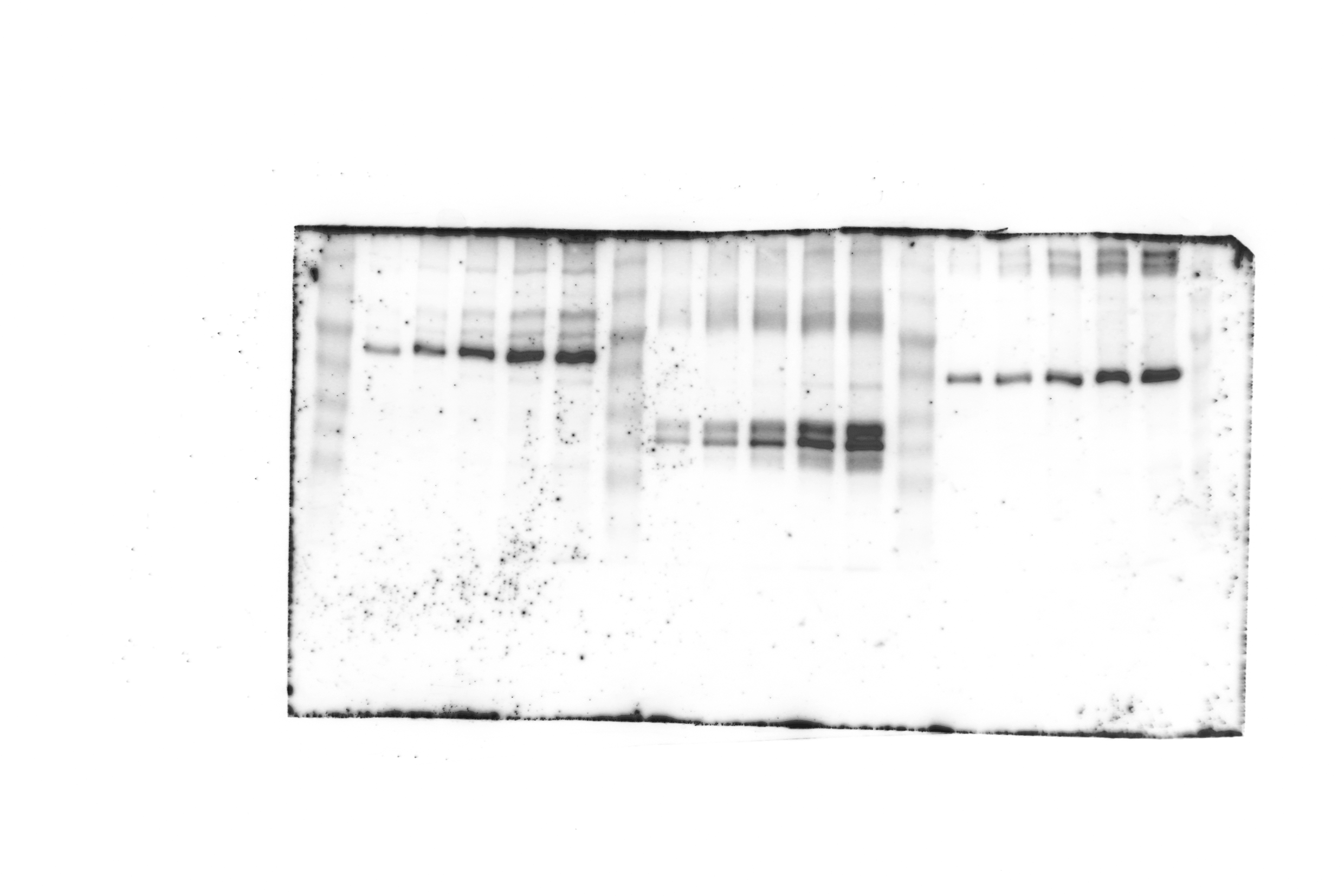

Supplement: Figure 5—source data 2. [file elife-77746-fig5-data2.zip › Fig.5/unedited/Fig.5A-FLAG(input).tif]

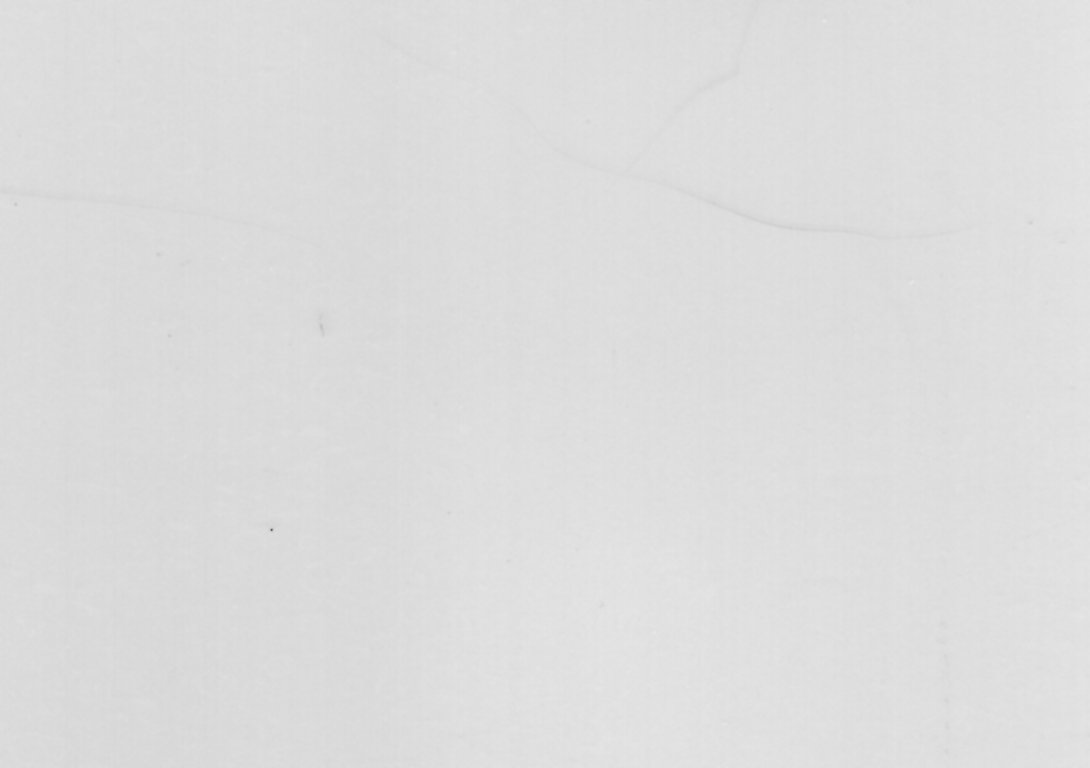

Supplement: Figure 5—source data 2. [file elife-77746-fig5-data2.zip › Fig.5/unedited/FIg.5F-FLAG(pulldown).tif]

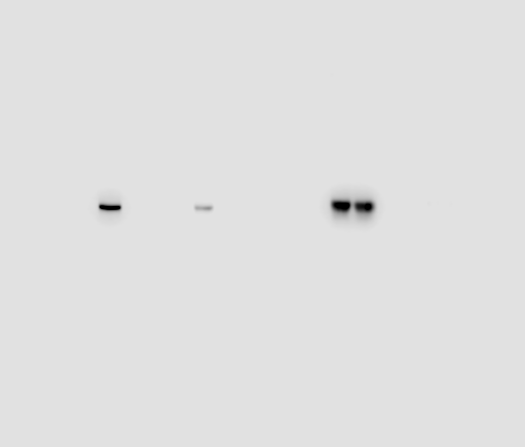

Supplement: Figure 5—source data 2. [file elife-77746-fig5-data2.zip › Fig.5/unedited/Fig.5B-FLAG&HA(IP).tif]

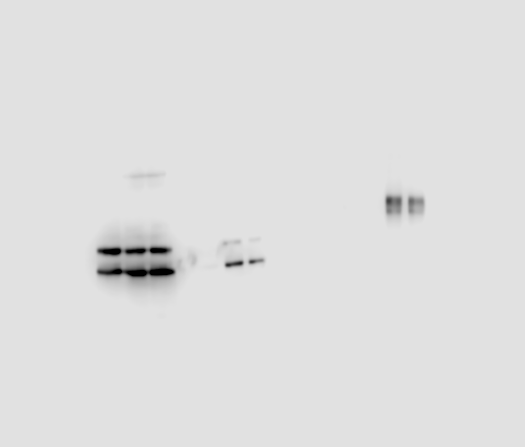

Supplement: Figure 5—source data 2. [file elife-77746-fig5-data2.zip › Fig.5/unedited/Fig.5B-HA(input).tif]

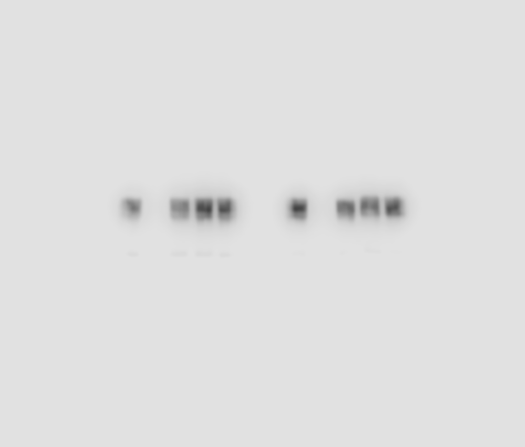

Supplement: Figure 5—source data 2. [file elife-77746-fig5-data2.zip › Fig.5/unedited/Fig.5F-HA(Myod input).tif]

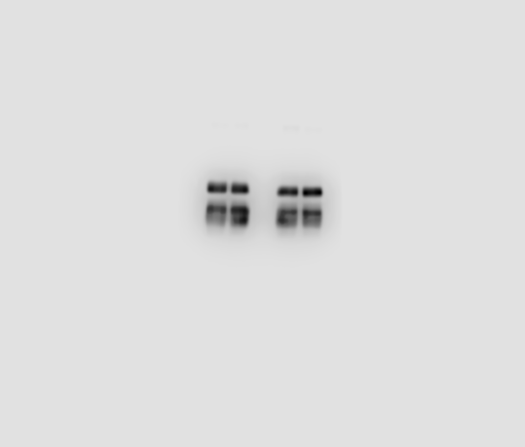

Supplement: Figure 5—source data 2. [file elife-77746-fig5-data2.zip › Fig.5/unedited/Fig.5E-myc(input).tif]

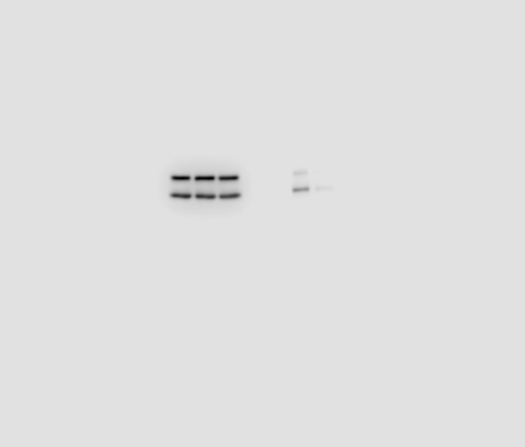

Supplement: Figure 5—source data 2. [file elife-77746-fig5-data2.zip › Fig.5/unedited/Fig.5B-myc(input).tif]

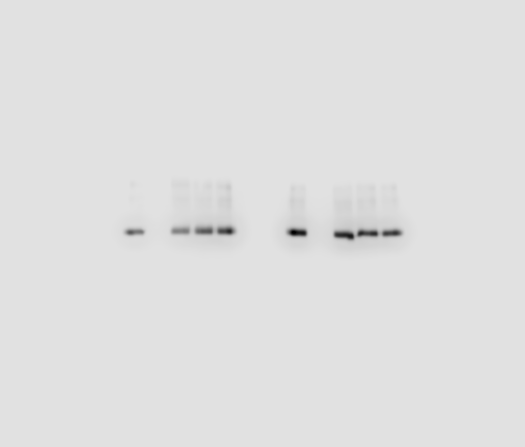

Supplement: Figure 5—source data 2. [file elife-77746-fig5-data2.zip › Fig.5/unedited/Fig.5F-HA(E47-input).tif]
