# Supplementary figures and images for: Actin-related protein 5 functions as a novel modulator of MyoD and MyoG in skeletal muscle and in rhabdomyosarcoma (part 2 of 2)
Source: eLife. 2022 Mar 29;11:e77746. doi: 10.7554/eLife.77746 (PMC8983046; doi:10.7554/eLife.77746)

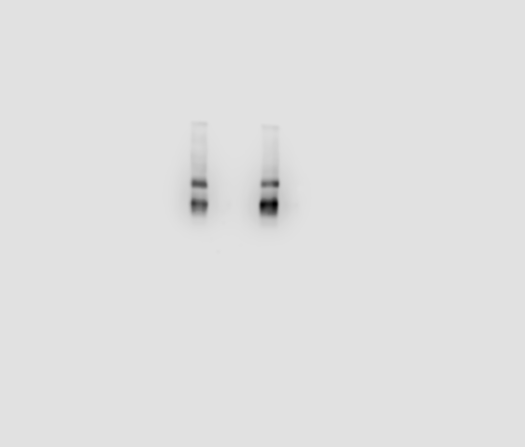

Supplement: Figure 5—source data 2. [file elife-77746-fig5-data2.zip › Fig.5/unedited/Fig.5E-myc(pulldown).tif]

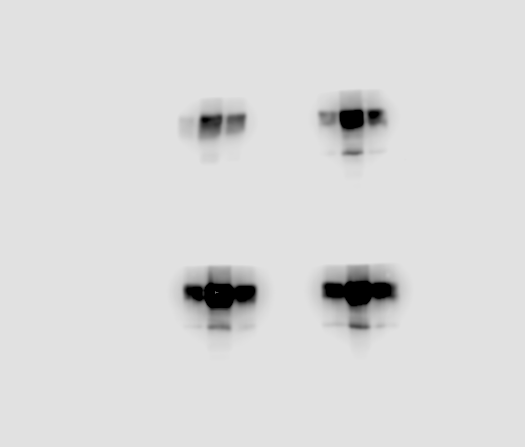

Supplement: Figure 5—source data 2. [file elife-77746-fig5-data2.zip › Fig.5/unedited/Fig.5F-HA(myoD PME1-pulldown.tif]

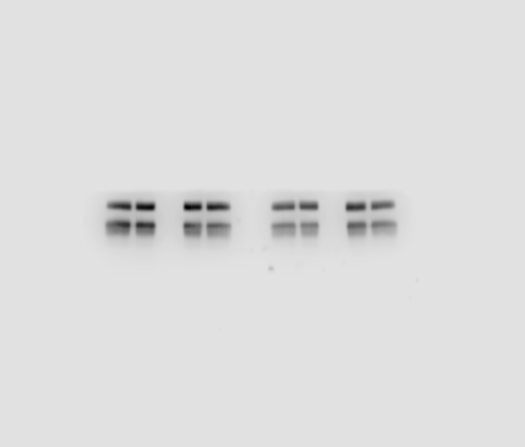

Supplement: Figure 5—source data 2. [file elife-77746-fig5-data2.zip › Fig.5/unedited/Fig.5F-myc(input).tif]

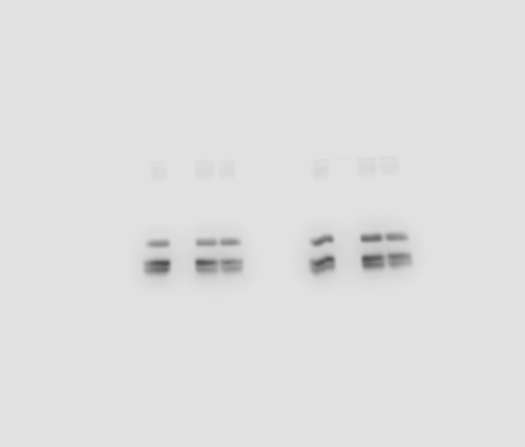

Supplement: Figure 5—source data 2. [file elife-77746-fig5-data2.zip › Fig.5/unedited/Fig.5F-myc(pulldown).tif]

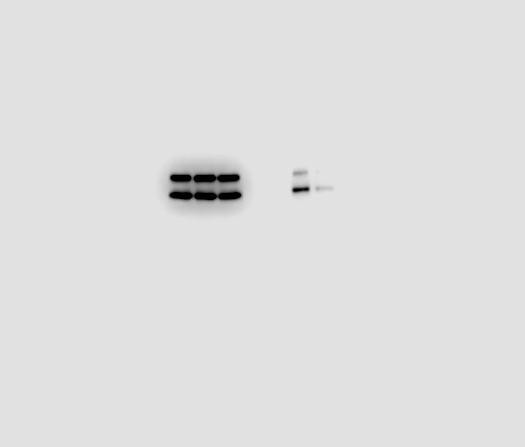

Supplement: Figure 5—source data 2. [file elife-77746-fig5-data2.zip › Fig.5/unedited/Fig.5B-myc(IP).tif]

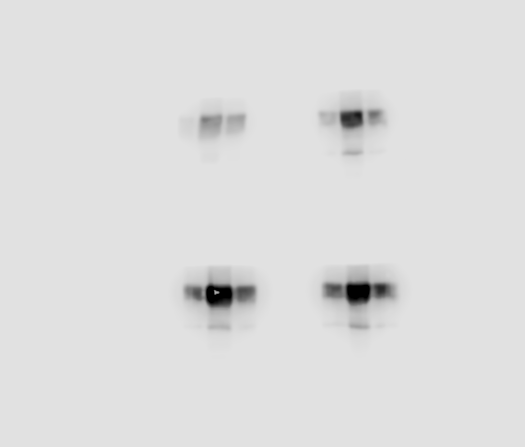

Supplement: Figure 5—source data 2. [file elife-77746-fig5-data2.zip › Fig.5/unedited/Fig.5F-HA(MyoD PME2-pulldown).tif]

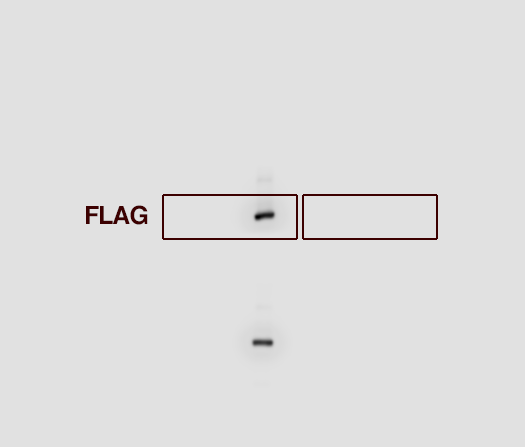

Supplement: Figure 6—source data 1. [file elife-77746-fig6-data1.zip › Fig.6/+label/Fig.6C-FLAG(+label).tif]

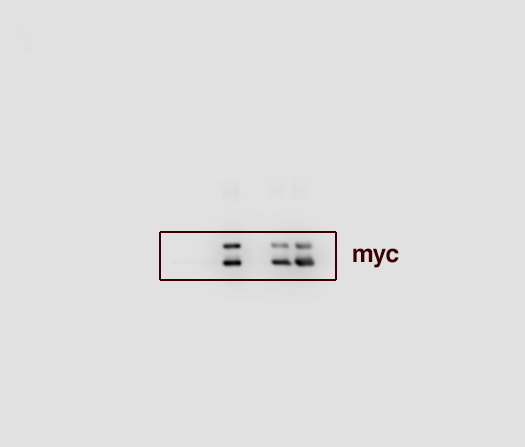

Supplement: Figure 6—source data 1. [file elife-77746-fig6-data1.zip › Fig.6/+label/Fig.6B-myc(pulldown)(+label).tif]

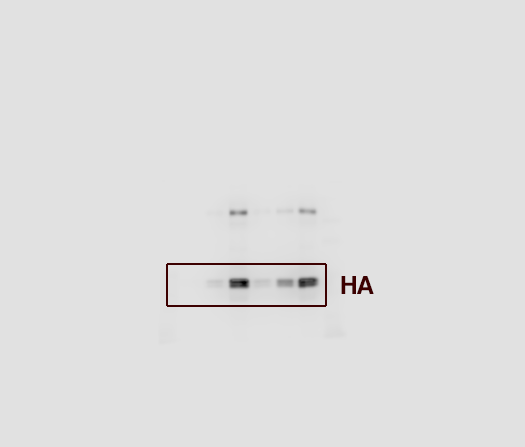

Supplement: Figure 6—source data 1. [file elife-77746-fig6-data1.zip › Fig.6/+label/Fig.6B-HA(MyoD-pulldown)(+label).tif]

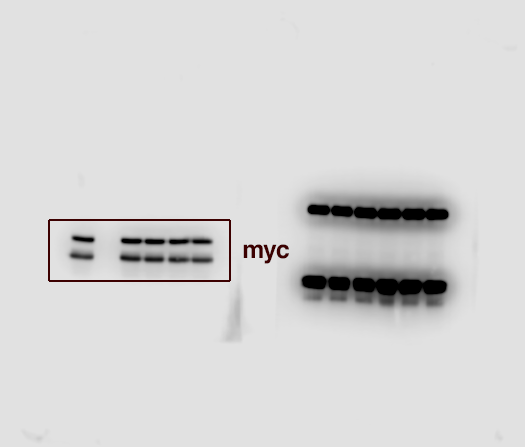

Supplement: Figure 6—source data 1. [file elife-77746-fig6-data1.zip › Fig.6/+label/Fig.6B-myc(input)(+label).tif]

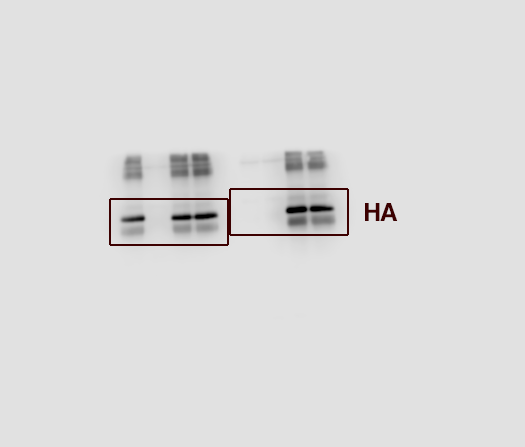

Supplement: Figure 6—source data 1. [file elife-77746-fig6-data1.zip › Fig.6/+label/Fig.6C-HA(+label).tif]

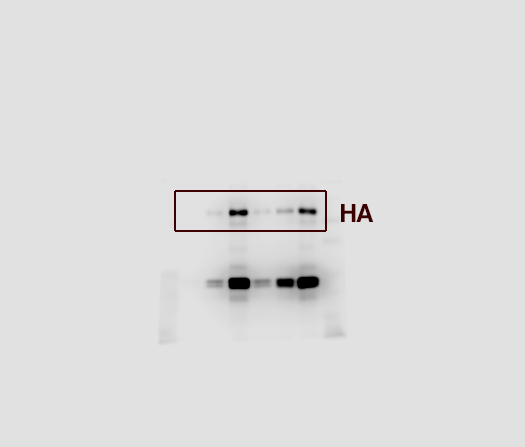

Supplement: Figure 6—source data 1. [file elife-77746-fig6-data1.zip › Fig.6/+label/Fig.6B-HA(E47-pulldown)(+label).tif]

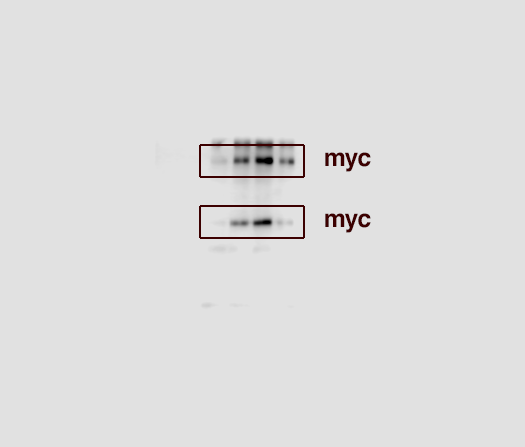

Supplement: Figure 6—source data 1. [file elife-77746-fig6-data1.zip › Fig.6/+label/Fig.6C-myc(pulldown)(+label).tif]

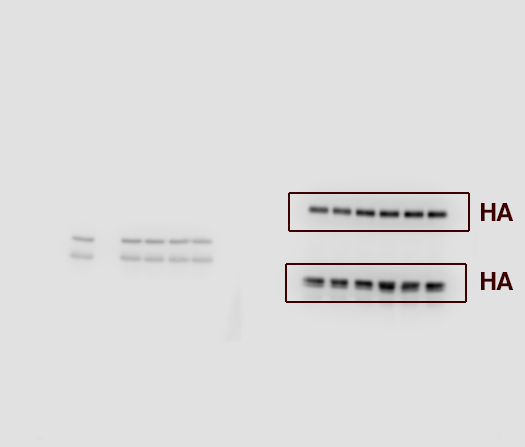

Supplement: Figure 6—source data 1. [file elife-77746-fig6-data1.zip › Fig.6/+label/Fig.6B-HA(input)(+label).tif]

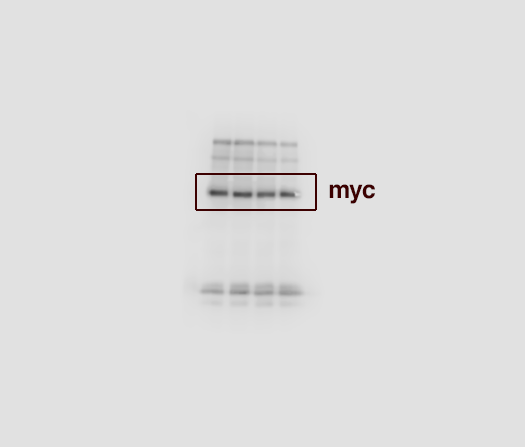

Supplement: Figure 6—source data 1. [file elife-77746-fig6-data1.zip › Fig.6/+label/Fig.6C-myc(E47-input)(+label).tif]

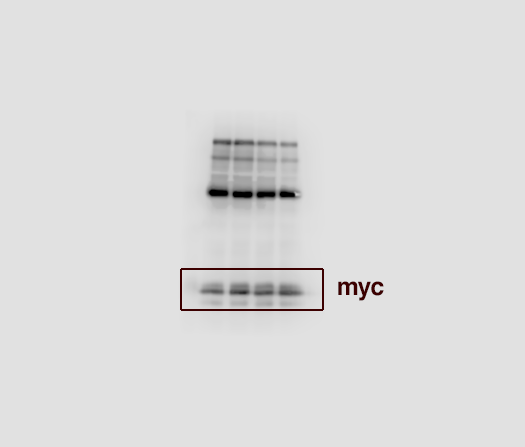

Supplement: Figure 6—source data 1. [file elife-77746-fig6-data1.zip › Fig.6/+label/Fig.6C myc(myog-input)(+label).tif]

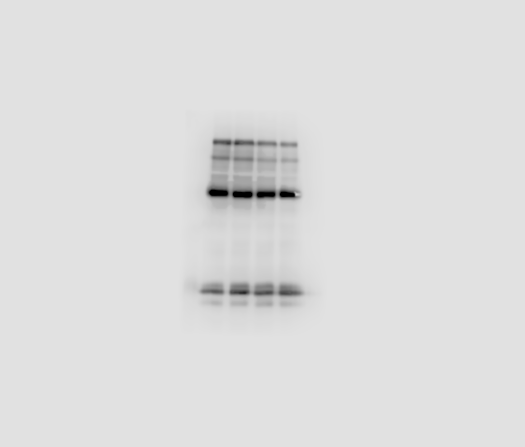

Supplement: Figure 6—source data 1. [file elife-77746-fig6-data1.zip › Fig.6/unedited/Fig.6C myc(myog-input).tif]

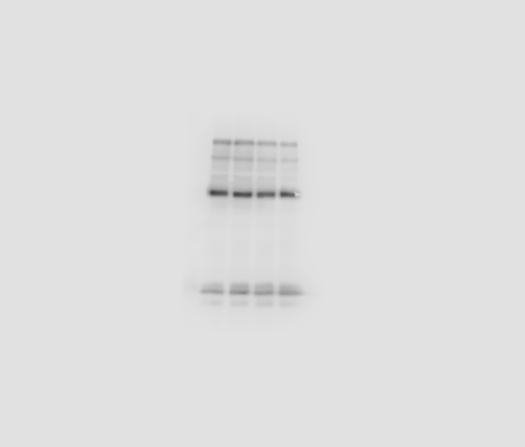

Supplement: Figure 6—source data 1. [file elife-77746-fig6-data1.zip › Fig.6/unedited/Fig.6C-myc(E47-input).tif]

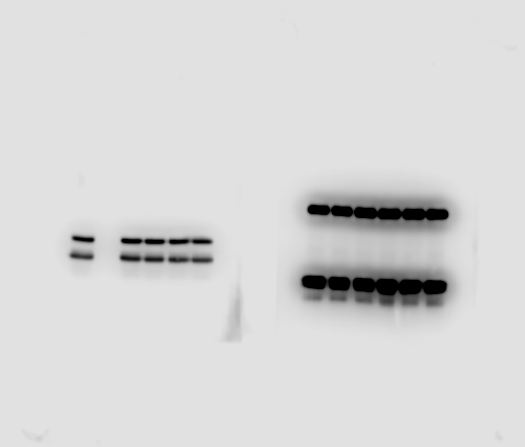

Supplement: Figure 6—source data 1. [file elife-77746-fig6-data1.zip › Fig.6/unedited/Fig.6B-myc(input).tif]

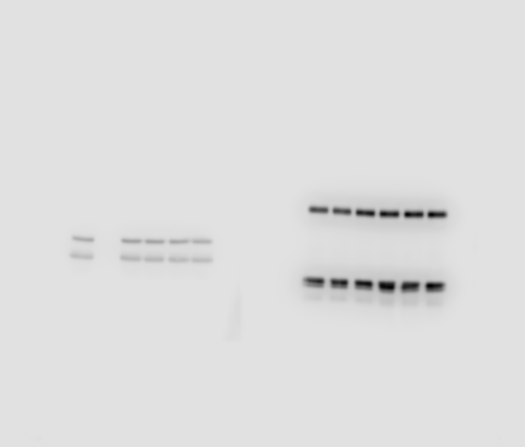

Supplement: Figure 6—source data 1. [file elife-77746-fig6-data1.zip › Fig.6/unedited/Fig.6B-HA(input).tif]

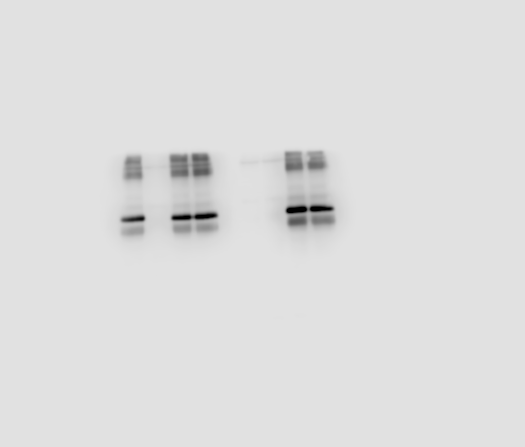

Supplement: Figure 6—source data 1. [file elife-77746-fig6-data1.zip › Fig.6/unedited/Fig.6C-HA.tif]

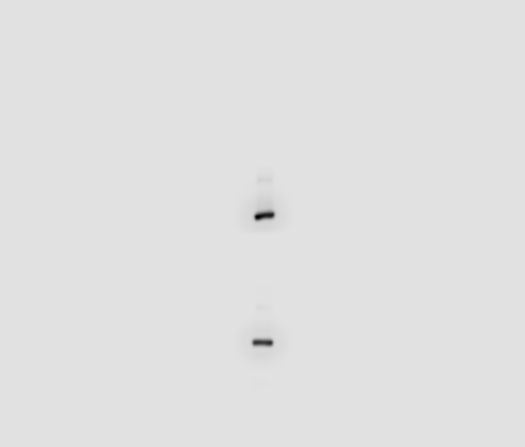

Supplement: Figure 6—source data 1. [file elife-77746-fig6-data1.zip › Fig.6/unedited/Fig.6C-FLAG.tif]

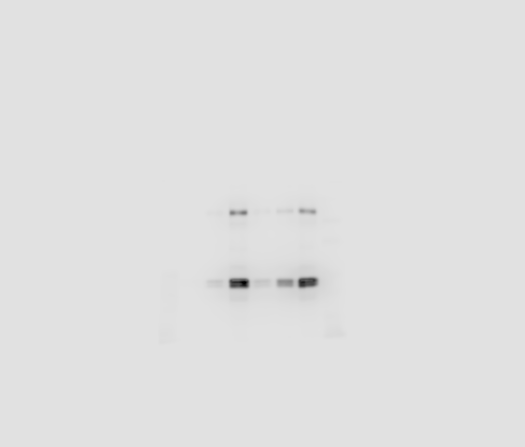

Supplement: Figure 6—source data 1. [file elife-77746-fig6-data1.zip › Fig.6/unedited/Fig.6B-HA(MyoD-pulldown).tif]

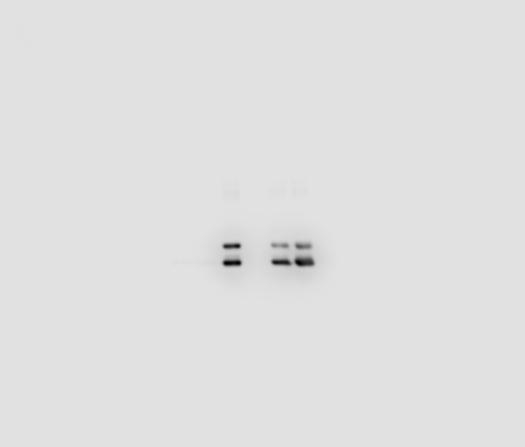

Supplement: Figure 6—source data 1. [file elife-77746-fig6-data1.zip › Fig.6/unedited/Fig.6B-myc(pulldown).tif]

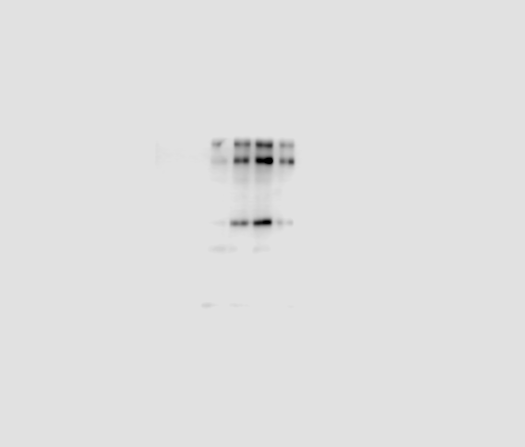

Supplement: Figure 6—source data 1. [file elife-77746-fig6-data1.zip › Fig.6/unedited/Fig.6C-myc(pulldown).tif]

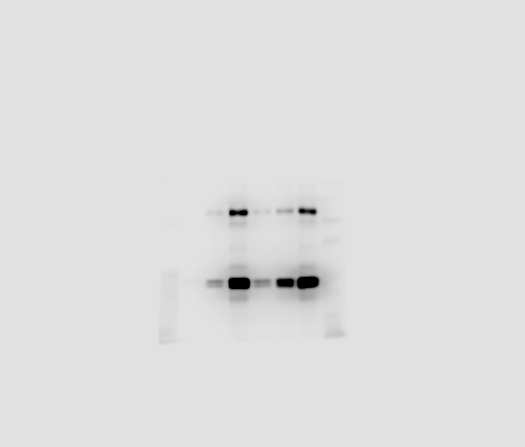

Supplement: Figure 6—source data 1. [file elife-77746-fig6-data1.zip › Fig.6/unedited/Fig.6B-HA(E47-pulldown).tif]
